# Supplementary material for: Network-based elucidation of colon cancer drug resistance mechanisms by phosphoproteomic time-series analysis
Source: Nat Commun. 2024 May 9;15:3909. doi: 10.1038/s41467-024-47957-3 (PMC11082183; doi:10.1038/s41467-024-47957-3)
Supplement: Supplementary file 1 — Supplemental Information [file 41467_2024_47957_MOESM1_ESM.pdf]

# Network-based elucidation of colon cancer drug resistance mechanisms by phosphoproteomic time-series analysis

## Supplemental Figures and Tables

George Rosenberger[1,†], Wenxue Li[2,†], Mikko Turunen[1,†], Jing He[1,3,†], Prem S Subramaniam[1], Sergey Pampou[1,4], Aaron T Griffin[1,5], Charles Karan[1,4], Patrick Kerwin[1], Diana Murray[1], Barry Honig[1,6,7,8], Yansheng Liu[2,9], Andrea Califano[1,4,6,7,10,11]

1 Department of Systems Biology, Columbia University Irving Medical Center, New York, NY, USA
2 Yale Cancer Biology Institute, Yale University, West Haven, CT, USA
3 Present address: Regeneron Genetics Center, Tarrytown, NY, USA
4 J.P. Sulzberger Columbia Genome Center, Columbia University Irving Medical Center, New York, NY, USA
5 Medical Scientist Training Program, Columbia University Irving Medical Center, New York, NY, USA
6 Department of Medicine, Columbia University Irving Medical Center, New York, NY, USA
7 Department of Biochemistry & Molecular Biophysics, Columbia University Irving Medical Center, New York, NY, USA
8 Zuckerman Mind Brain and Behavior Institute, Columbia University, New York, NY, USA
9 Department of Pharmacology, Yale University School of Medicine, New Haven, CT, USA
10 Herbert Irving Comprehensive Cancer Center, Columbia University Irving Medical Center, New York, NY, USA
11 Department of Biomedical Informatics, Columbia University Irving Medical Center, New York, NY, USA
† Equal Contribution

Correspondence to: yansheng.liu@yale.edu and ac2248@cumc.columbia.edu

# Supplemental Figures

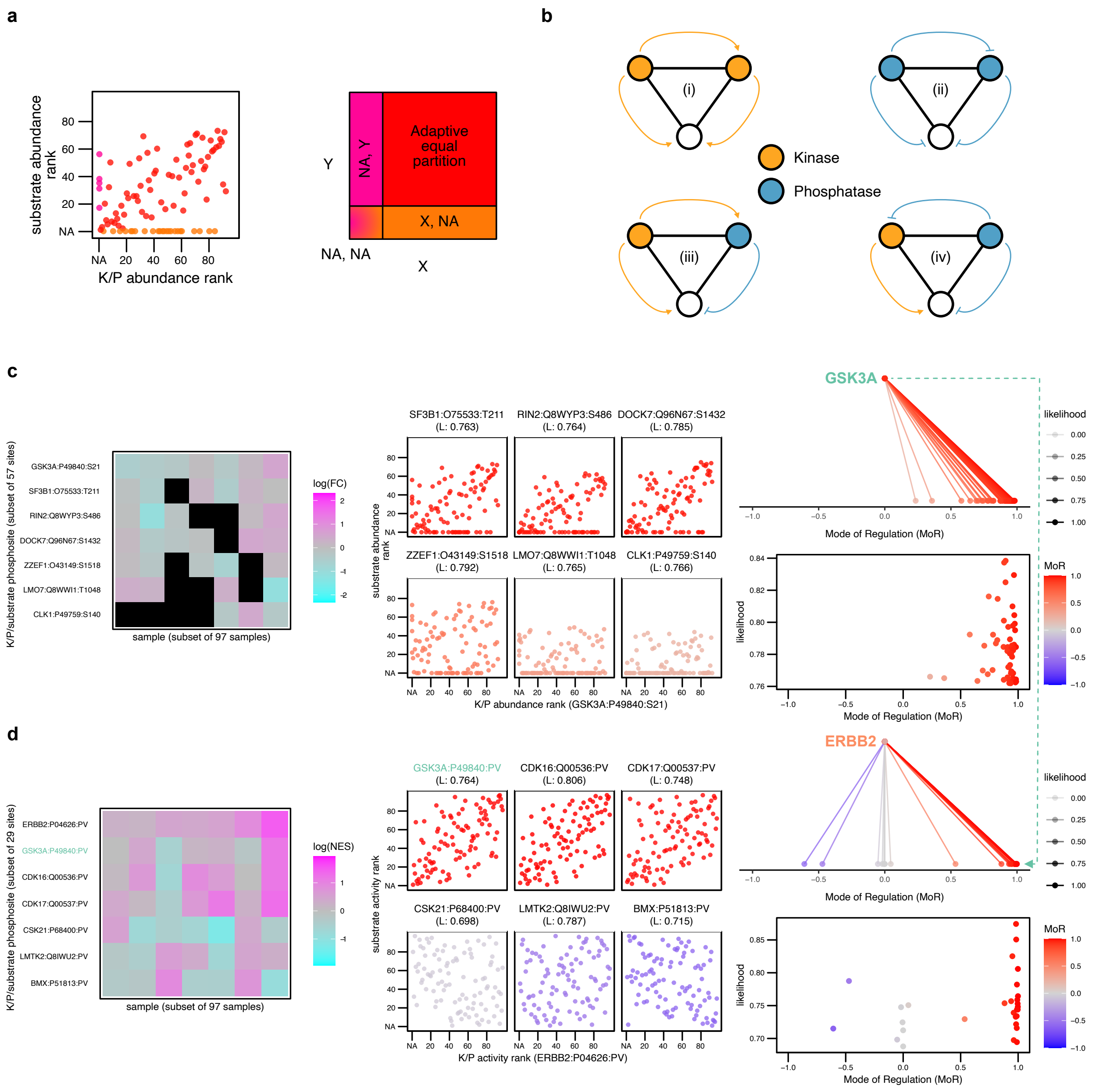


**Supplemental Figure 1. Walkthrough example of VESPA. a** Mutual Information (MI) is assessed by a Hybrid Partitioning Estimator, which splits datapoints into four quadrants, depending on whether both KP-enzyme X and substrate Y were measured (adaptive equal partition), only X or Y where measured (X, NA; NA, Y), or neither X nor Y were measured (NA, NA) in the samples (Methods). **b** The signal transduction Data Processing Inequality (stDPI) accounts for a limited set of enzymatic reactions supported by kinases and phosphatases more likely to be measurable by serine/threonine-enriched phosphoproteomics (see Methods). The goal of this approach is to model the possible

indirect interactions between a KP-enzyme and its candidate substrates (Methods): Only the following indirect KP → KP → S interactions are considered for stDPI analysis, including K → K → S (i) or P → K → S (iv). Because P → P → S (ii) and K → P → S (iii) interactions will result in conflicting substrate phosphorylation *vs.* dephosphorylation compared with the alternative direct interactions, the conditions required for evaluation of stDPI are not fulfilled (see Methods). **c** Illustrative example of an individual phosphostate-level VESPA analysis, including generation of the raw phosphoproteomic data matrix, mutual information and probabilistic weight estimation using the phosphopeptide abundance of the kinase GSK3A and of candidate targets SF3B1, RIN2, DOCK7, ZZEF1, LMO7, and CLK1 as examples (including accounting for missing values). Signalons are visualized, including Mode of Regulation (MoR) and probabilistic weight. Signalon substrates include only activated and inactivated substrates for kinases and phosphatases, respectively. **d** Example of activity-level analysis shows how results from dVESPA analysis at the phosphostate level are used to infer KP-enzyme activity based on substrate activity. As an illustrative example, the matrix on the left represents the quantitative assessment of ERBB2's substrate activity, as inferred from dVESPA analysis of their own substrates' phosphostate. MI is computed between the phosphostate of a KP-enzyme (ERBB2 in this case) and the activity of its candidate substrates.

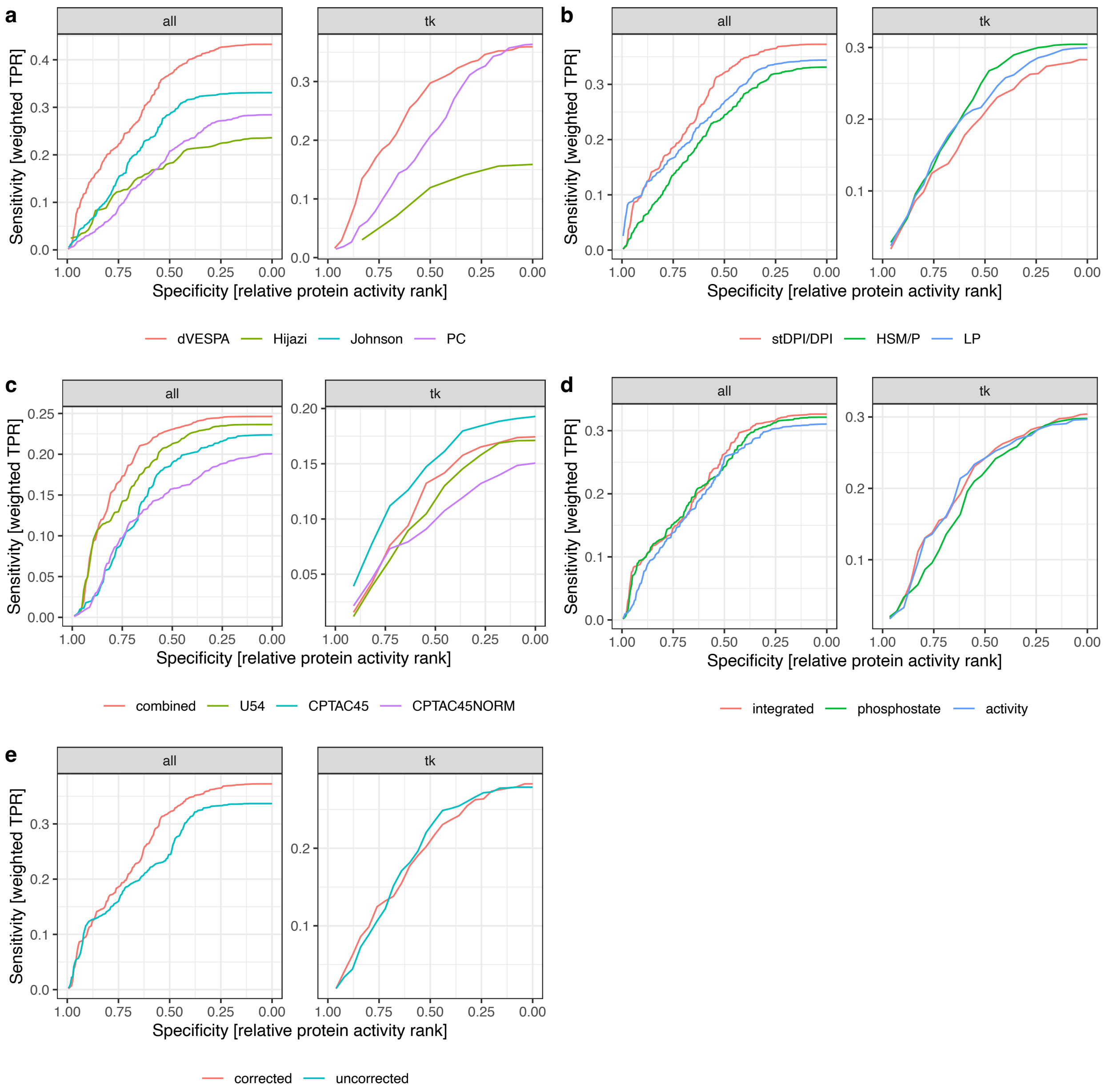


**Supplemental Figure 2. mVESPA Benchmarking (intersection signalon sets).** mVESPA was benchmarked using the GDSC drug sensitivity dataset, comparing the effects of different K/P-S networks and algorithmic improvements on predictive performance. For each differential comparison, ROC metrics were computed as described previously (see Methods), where the sensitivity represents the mVESPA scores, weighted by GDSC drug sensitivity, and the selectivity represents a normalized rank of the top VESPA hits (see Methods). The individual ROC curves were then averaged. Statistical comparison of the differential comparison AUC metrics was conducted using an unpaired, right tailed Wilcox' tests. For each comparison, signalons were limited to the set of K/P-enzymes covered by all other signalons

(intersection), thus allowing to compare the selectivity of the different approaches. Area-under-the-curve (AUC) and *p*-value metrics are reported in Supplemental Tables 1 and 2, respectively. The following comparative analyses were performed independently for all kinases (ALL) or only tyrosine kinases (TK): **a** Using context-specific *vs.* reference-based signalons, **b** using of dVESPA-inferred signalons with stDPI/DPI *vs.* reference-guided signalons (Hierarchical statistical model (HSM); LinkPhinder (LP), **c** Leveraging signalon integration and optimization across multiple datasets, **d** Leveraging hierarchical integration and **e** introducing mVESPA crosstalk correction. As shown, each independent enhancement introduced in VESPA improves its performance, either across all kinases or for TKs. Source data are provided as a Source Data file.

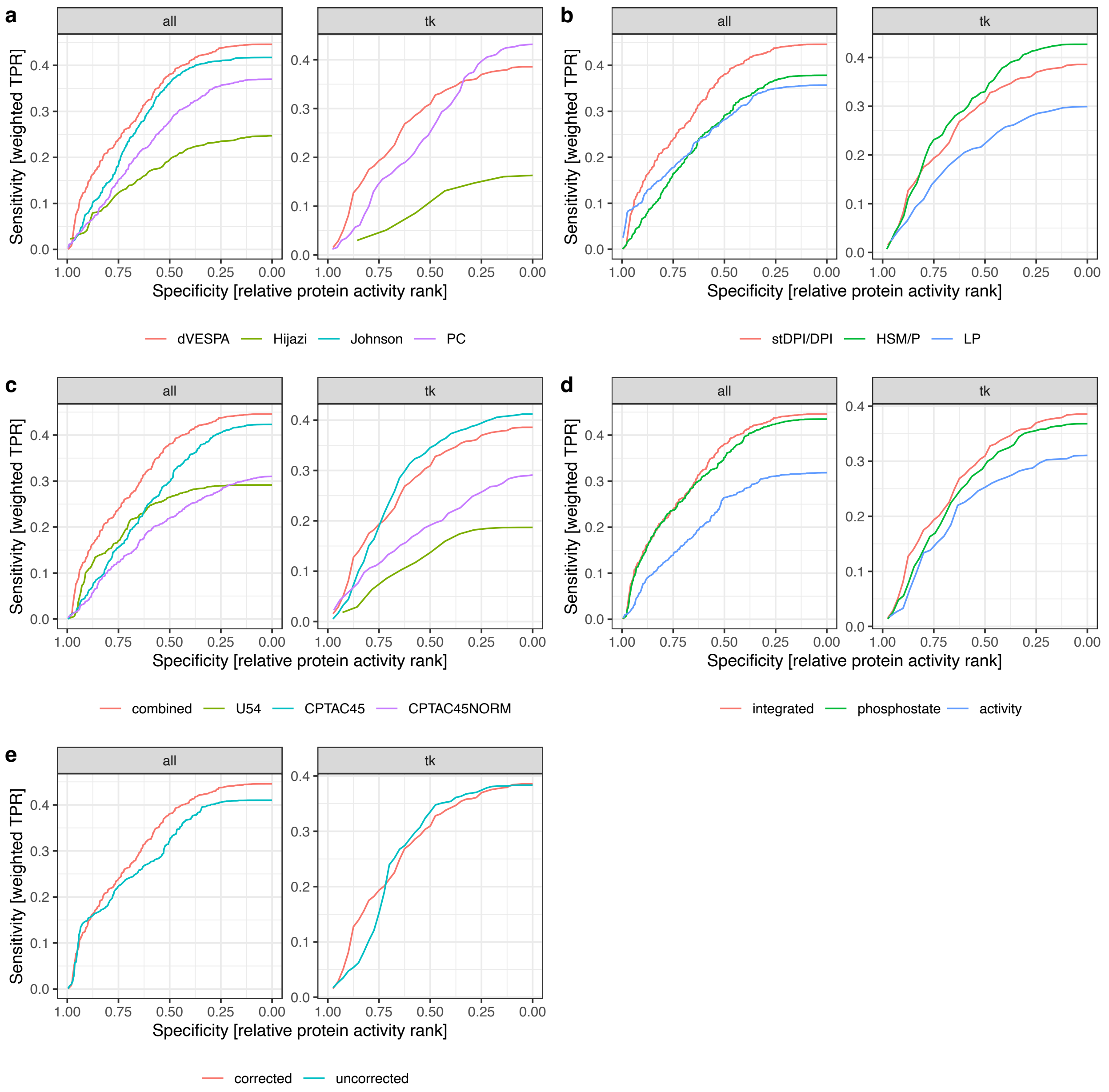


**Supplemental Figure 3. mVESPA benchmarking (full signalon sets).** mVESPA was benchmarked using the GDSC drug sensitivity dataset, comparing the effects of different K/P-S networks and algorithmic improvements on predictive performance. The same metrics as shown in Supplemental Figure 2 are evaluated but the signalons were not limited to the intersection of K/P-enzymes covered by all reference networks, thus allowing to compare the sensitivity of the different approaches. For each differential comparison, ROC metrics were computed as described previously (see Methods), where the sensitivity represents the mVESPA scores, weighted by GDSC drug sensitivity, and the selectivity represents a normalized rank of the top VESPA hits (see Methods). The individual ROC curves were then averaged. Statistical comparison of the differential comparison AUC metrics was conducted using an unpaired, right tailed Wilcox'

tests. For each comparison, signalons were limited to the set of K/P-enzymes covered by all other signalons (intersection), thus allowing to compare the selectivity of the different approaches. Area-under-the-curve (AUC) and *p*-value metrics are reported in Supplemental Tables 1 and 2, respectively. The following comparative analyses were performed independently for all kinases (ALL) or only tyrosine kinases (TK): **a** Using context-specific *vs.* reference-based signalons, **b** using of dVESPA-inferred signalons with stDPI/DPI *vs.* reference-guided signalons (Hierarchical statistical model (HSM); LinkPhinder (LP), **c** Leveraging signalon integration and optimization across multiple datasets, **d** Leveraging hierarchical integration and **e** introducing mVESPA crosstalk correction. As shown, each independent enhancement introduced in VESPA improves its performance, either across all kinases or for TKs. Source data are provided as a Source Data file.

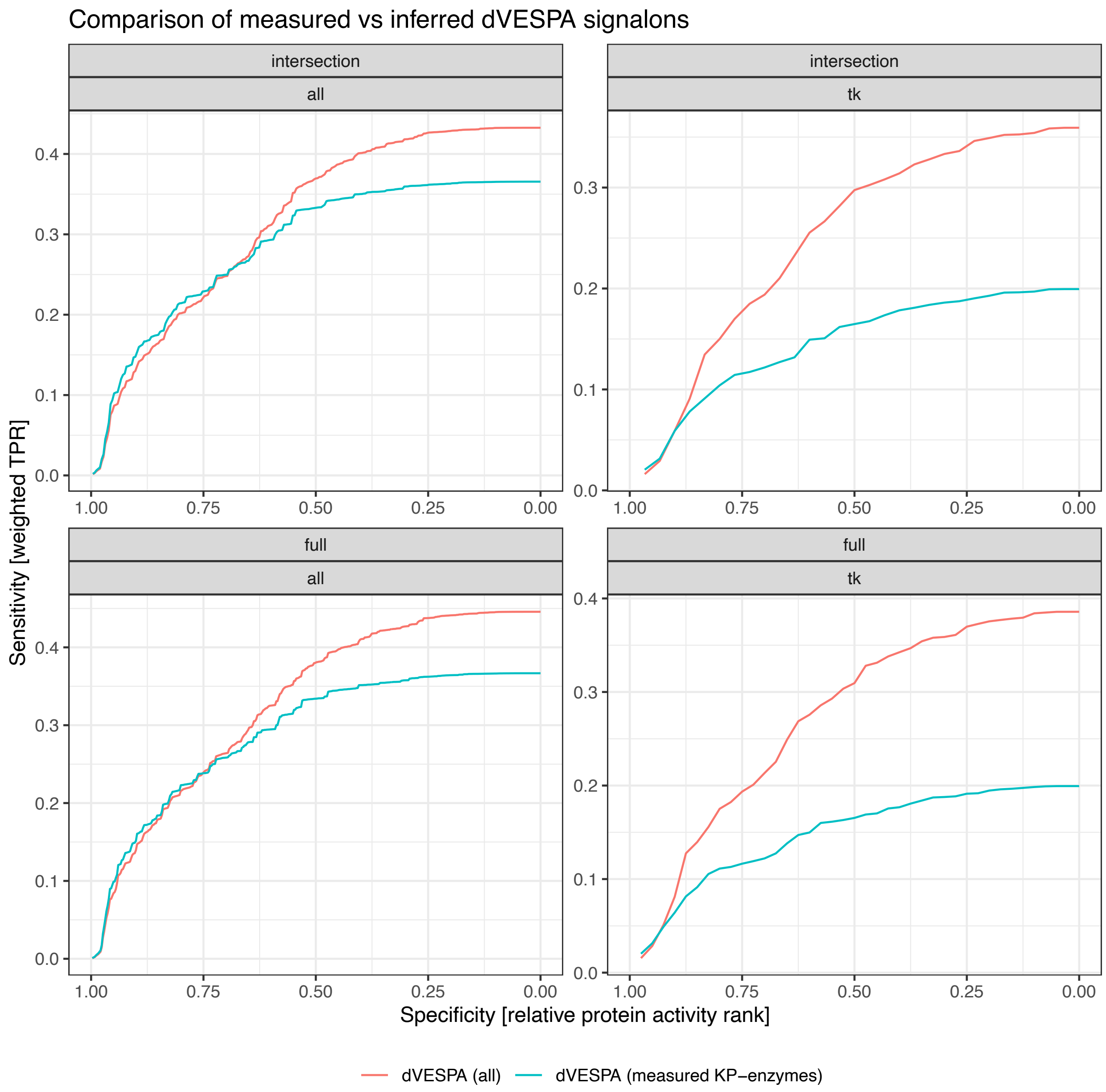


**Supplemental Figure 4. Comparison of dVESPA signalons.** Either all signalons are used or only those where phosphopeptides of the corresponding K/P-enzyme were measured by phosphoproteomics. The same metrics as shown in Supplemental Figure 2 are evaluated. Source data are provided as a Source Data file.

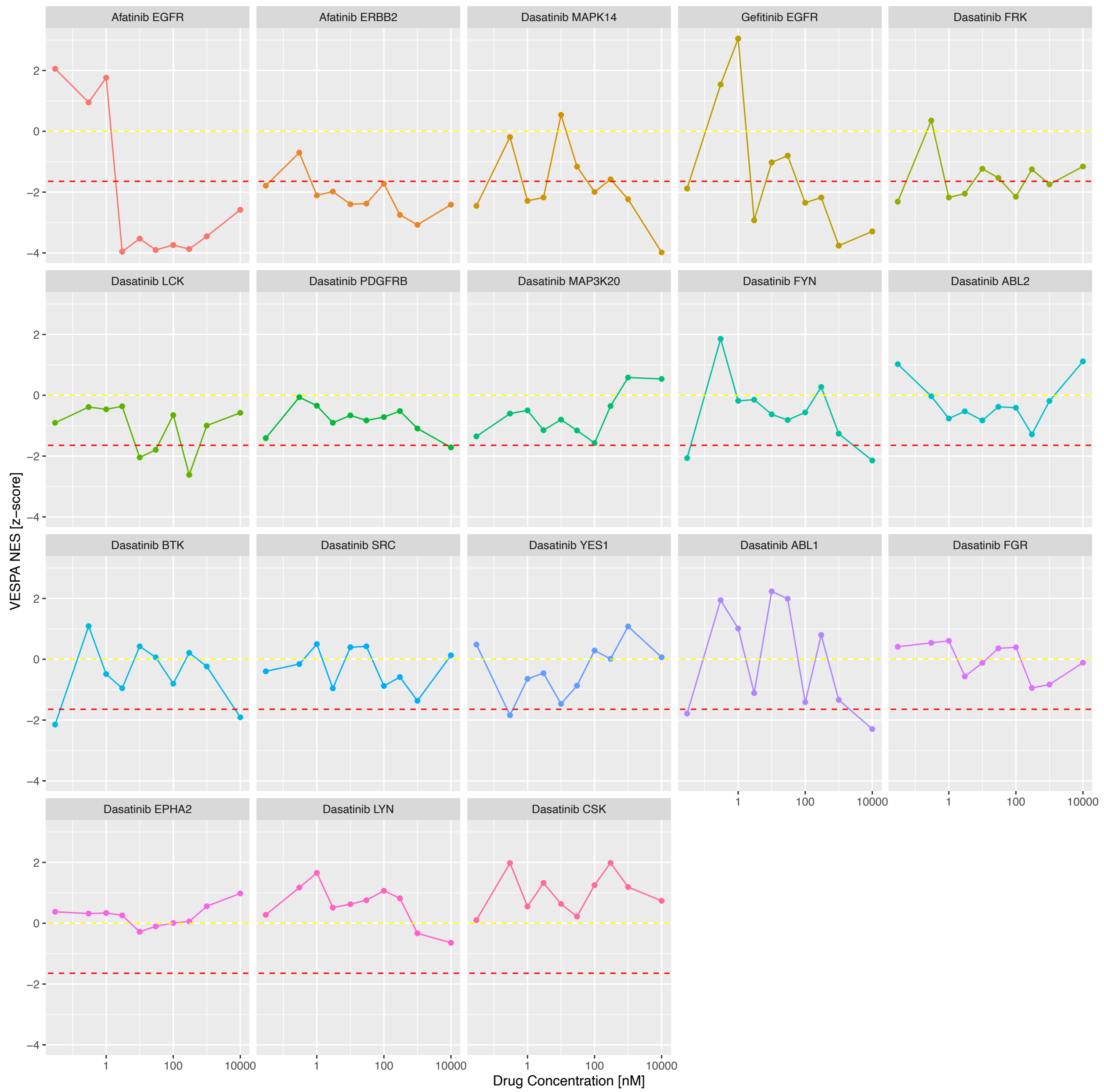


**Supplemental Figure 5. Application of VESPA to decryptM dataset.** Using CPTAC-derived signalons for LSCC, VESPA was applied to ten-point drug dosage series of Afatinib, Gefitinib and Dasatinib. The main drug targets identified by DrugBank and covered by VESPA are depicted. Negative VESPA NES (z-score) values indicate inhibition of kinase activity, where a value of NES < -1.65 ($p < 0.05$; red line) is significant. Entries are ordered according to increasing median z-score. Source data are provided as a Source Data file.

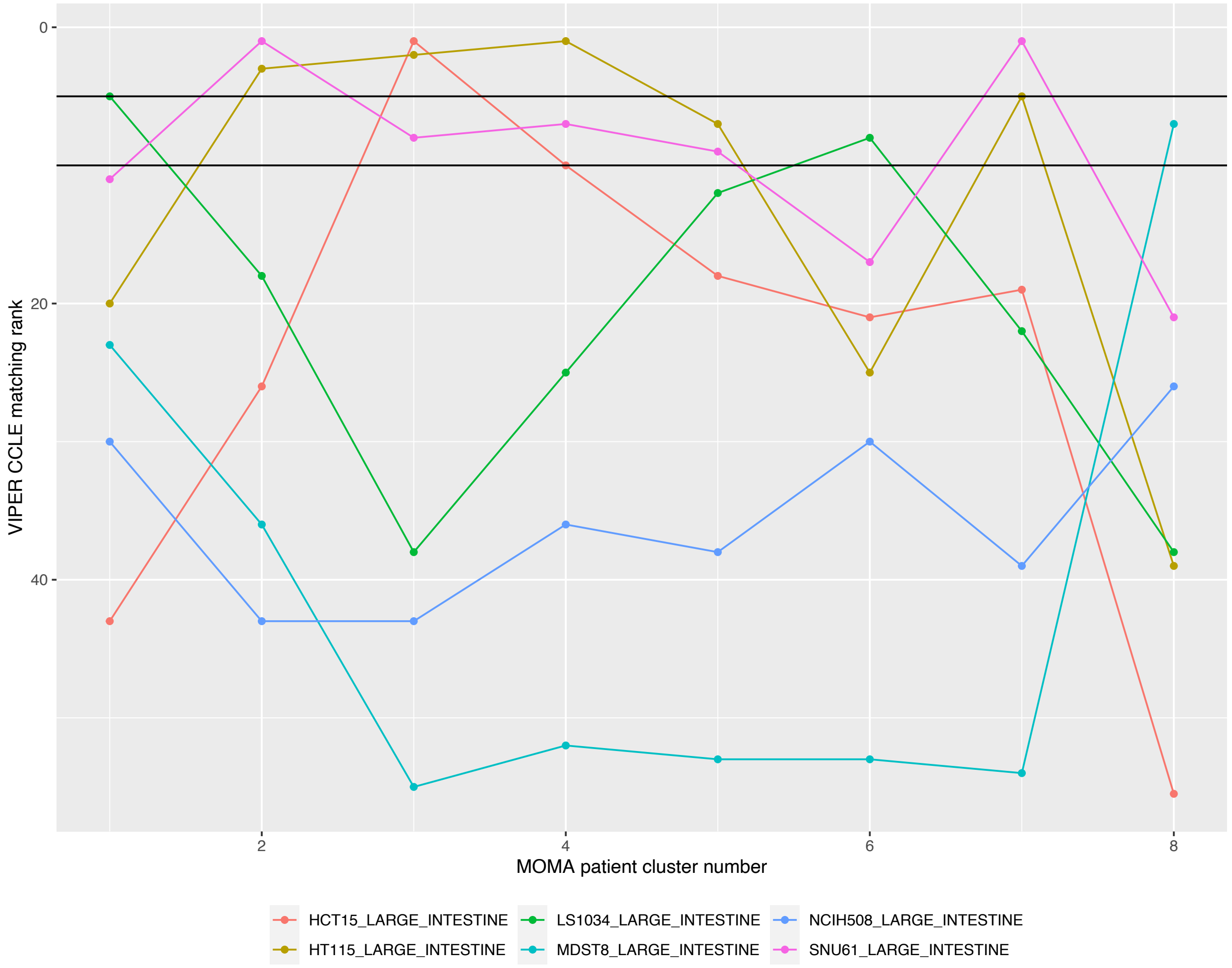


**Supplemental Figure 6. Representative CRC cell line selection.** For each CRC cluster determined by MOM analysis of the TCGA CRC cohort, CRC CCLE cell lines were ranked according to the enrichment of their 50 most differentially active proteins in proteins differentially active in each cluster, based on Stouffer integration of their differential activity in each cluster sample (Methods). Source data are provided as a Source Data file.

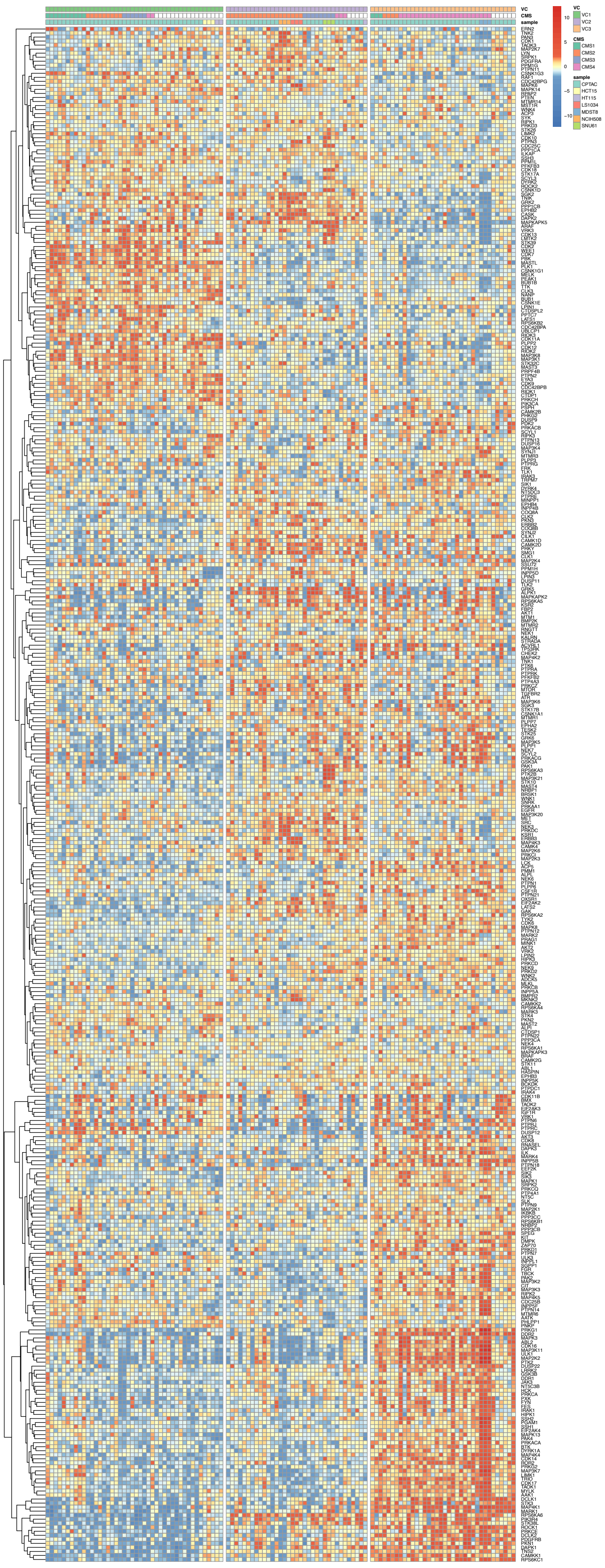
VC
CMS
sample
10
5
0
−5
−10
VC
VC1
VC2
VC3
CMS
CMS1
CMS2
CMS3
CMS4
sample
CPTAC
HCT15
HT115
LS1034
MDST8
NCIH508
SNU61

**Supplemental Figure 7. VESPA matrix sorted by VC cluster identity.** KP-enzymes and their VESPA inferred differential activity (i.e., normalized enrichment scores, NES) across all CRC CPTAC tumor samples and unperturbed cell lines for which phosphoproteomic profile data was available. CPTAC clinical profiles and cell lines were grouped according to the Consensus Molecular Classifier (CMS) and VESPA clusters (VC). The samples are sorted according to VC classification. Source data are provided as a Source Data file.

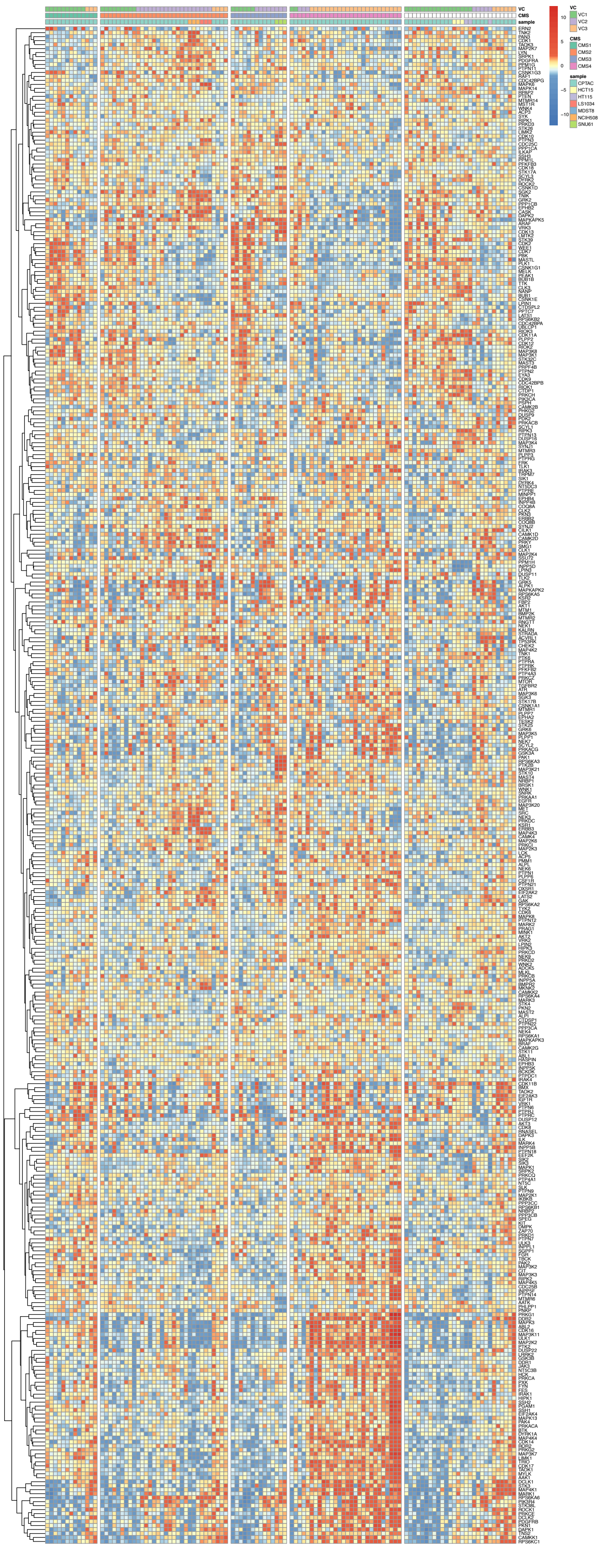
VC
CMS
sample
10
5
0
-5
-10
VC
VC1
VC2
VC3
CMS
CMS1
CMS2
CMS3
CMS4
sample
CPTAC
HCT15
HT115
LS1034
MDST8
NCIH508
SNU61

**Supplemental Figure 8. VESPA matrix sorted by CMS cluster identity.** Same as Supplemental Figure 7 but with samples sorted according to CMS classification. Source data are provided as a Source Data file.

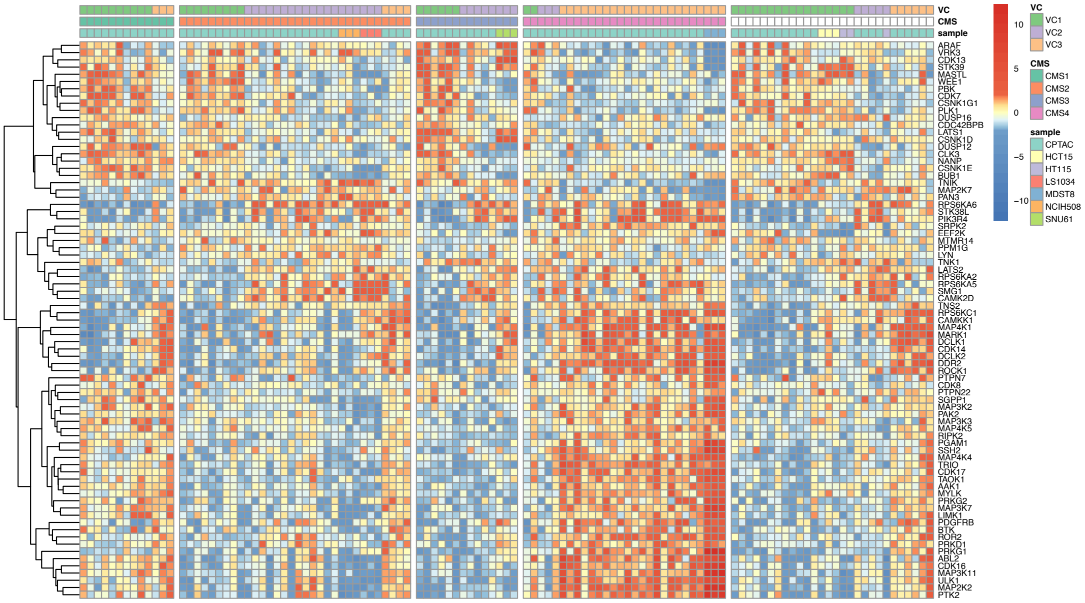


**Supplemental Figure 9. VESPA matrix: most informative proteins.** Same as Supplemental Figure 7 but showing only the most informative proteins for CMS classification, sorted by CMS cluster identity (Methods). Source data are provided as a Source Data file.

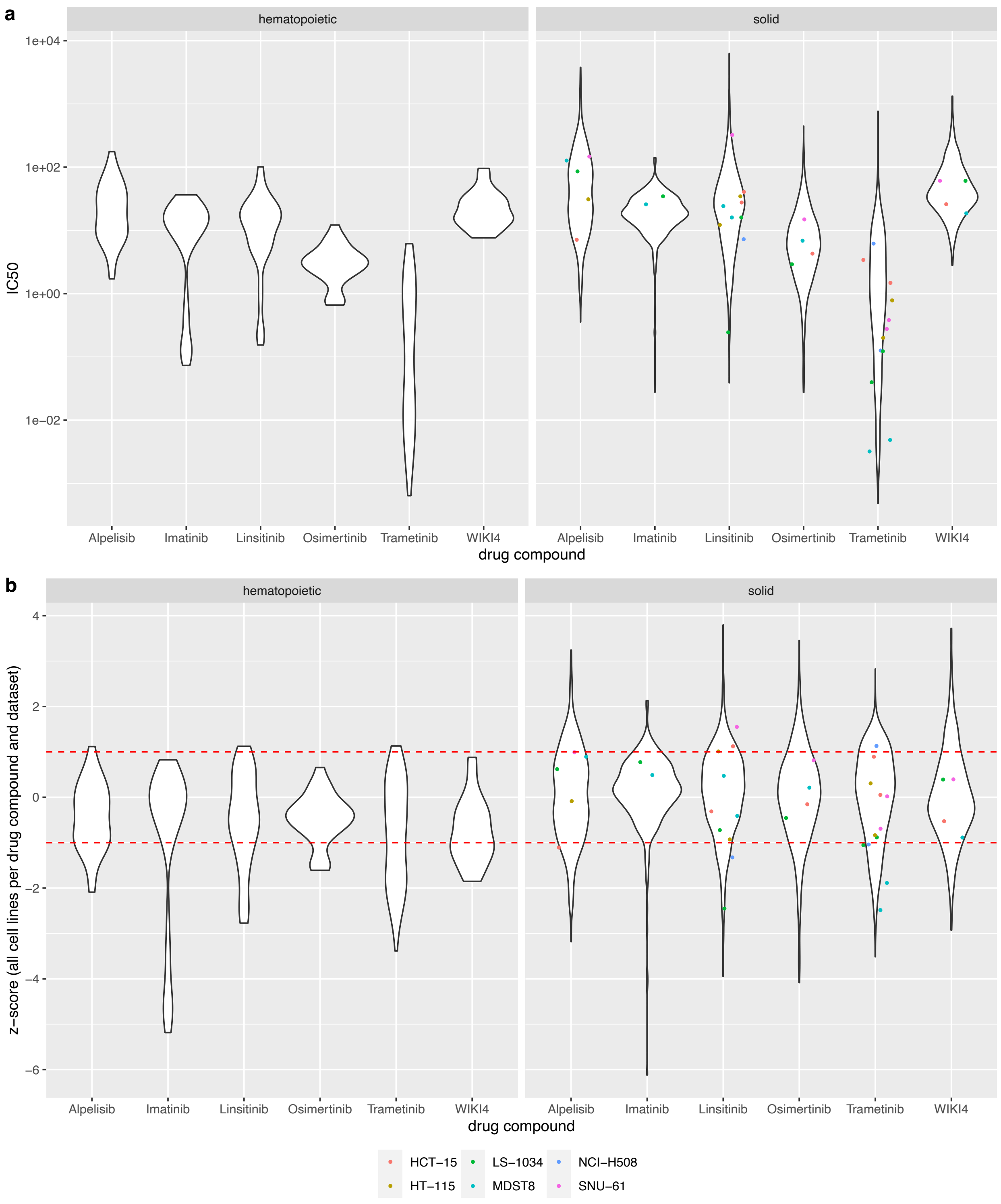


**Supplemental Figure 10. Sensitivity of selected cell lines to drug panel as reported in GDSC. A.** Violin plots show the distribution of GDSC 1&2 IC50 values in each cell line for each drug in the panel (Methods). **B.** Violin plots show the distribution of GDSC 1&2 *z*-score values in each cell line, for each drug in the panel (Methods). For each drug,

n = 33, 39, 69, 27, 64, and 27 datapoints representing distinct cell lines were used to draw the hematopoietic tumor distribution, while n = 676, 325, 1417, 636, 1412, and 635 datapoints, respectively were used to draw the solid tumor distribution. Red lines indicate thresholds used to select sensitive and resistant cell lines (z-score < -1.0 and > 1.0, respectively). Source data are provided as a Source Data file.

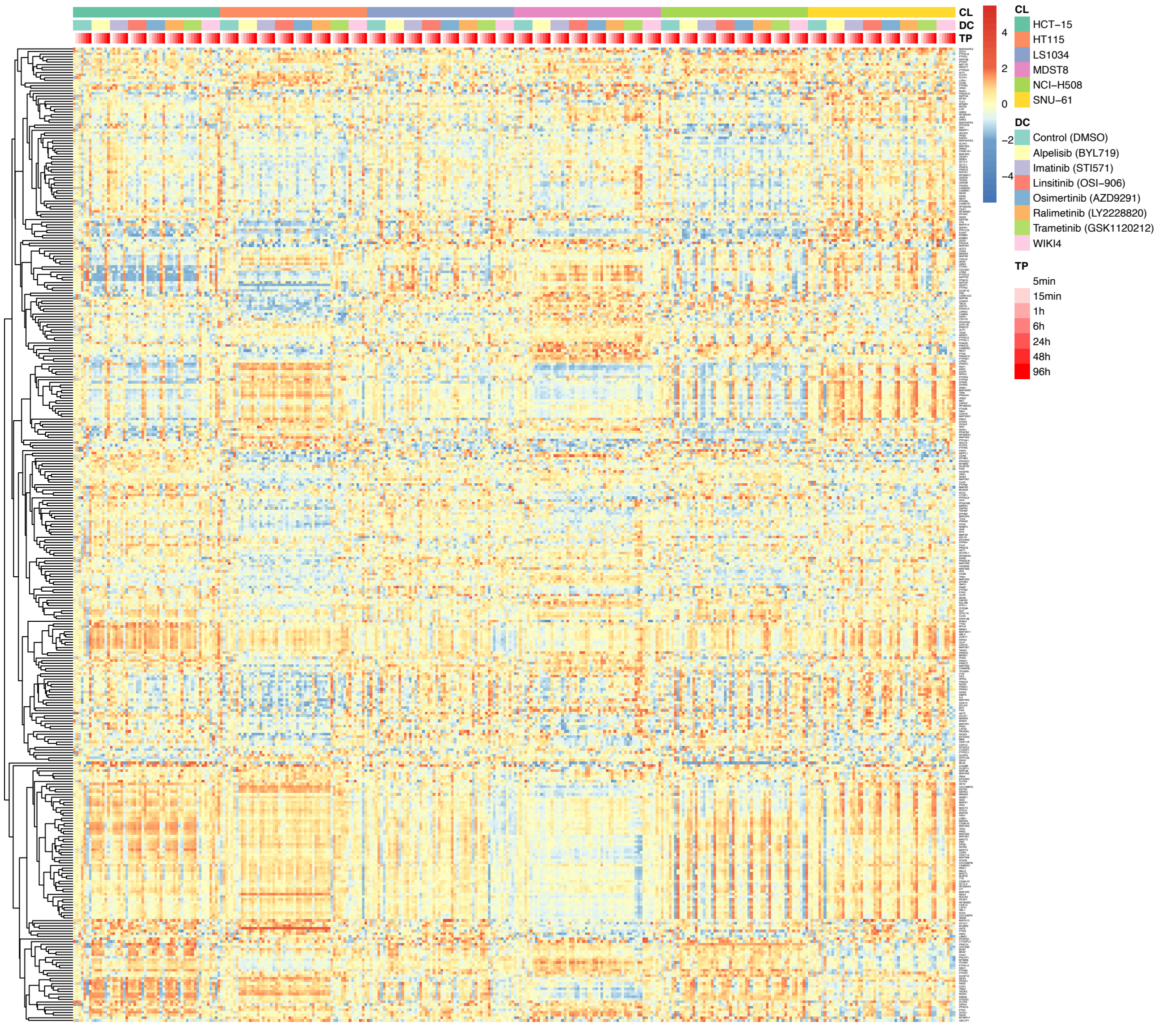


**Supplemental Figure 11. KP-enzyme activity by phosphostate-level VESPA analysis.** Matrix representing the differential activity of all KP-enzyme assessed by phosphostate-level VESPA analysis across all drug treated cell lines (336 samples), covering six CRC cell lines, 7 drug perturbations and DMSO control across 7 time points. Samples are sorted progressively by cell line, drug treatment, and time point. KP-enzymes are organized by hierarchical cluster analysis. Source data are provided as a Source Data file.

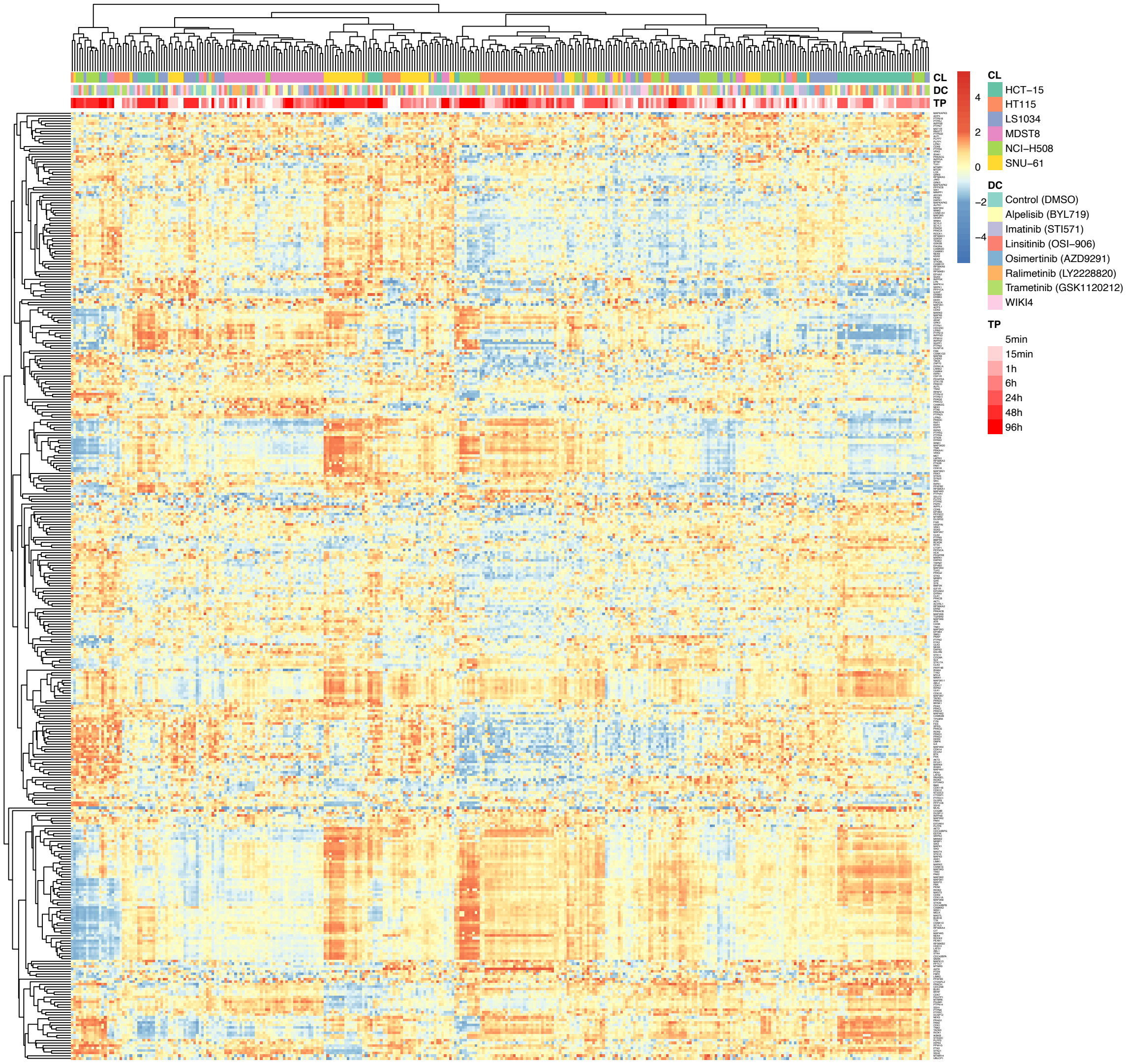


**Supplemental Figure 12. KP-enzyme activity by phosphostate-level VESPA analysis (clustered).** Same as Supplemental Figure 11 but with both samples and KP-enzymes organized by hierarchical cluster analysis. Source data are provided as a Source Data file.

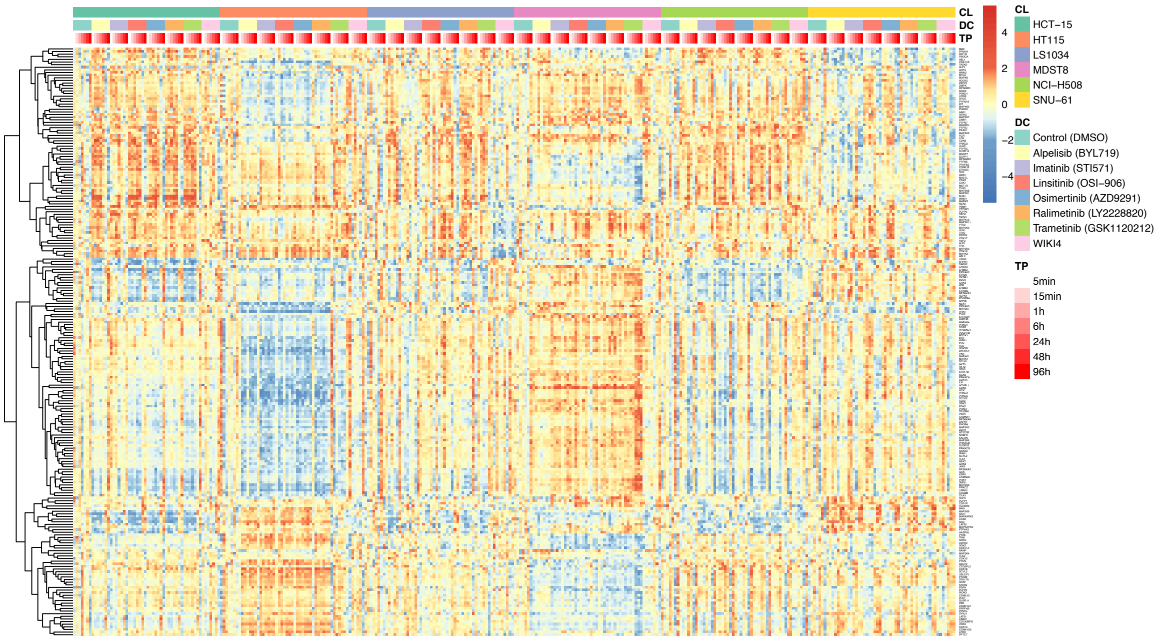


**Supplemental Figure 13. KP-enzyme activity by activity-level VESPA analysis.** Same as Supplemental Figure 11 but showing differential KP-enzyme activity based on activity-level VESPA analysis. Source data are provided as a Source Data file.

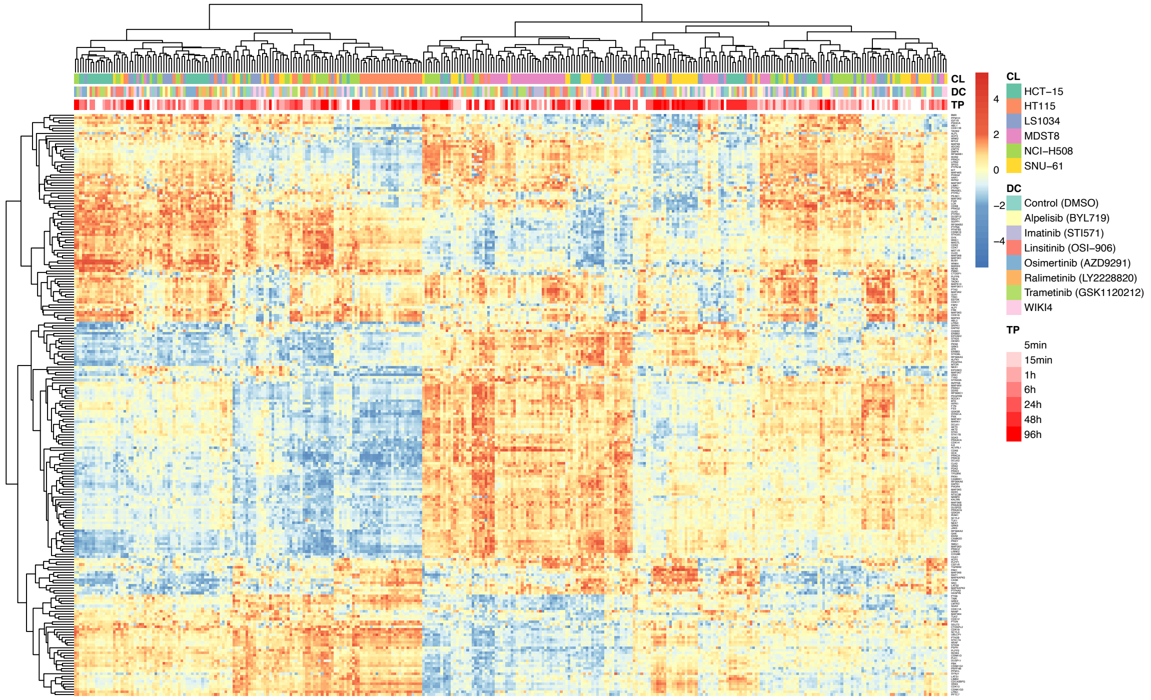


**Supplemental Figure 14. KP-enzyme activity by activity-level VESPA analysis (clustered).** Same as Supplemental Figure 12 but showing differential KP-enzyme activity based on activity-level VESPA analysis. Source data are provided as a Source Data file.

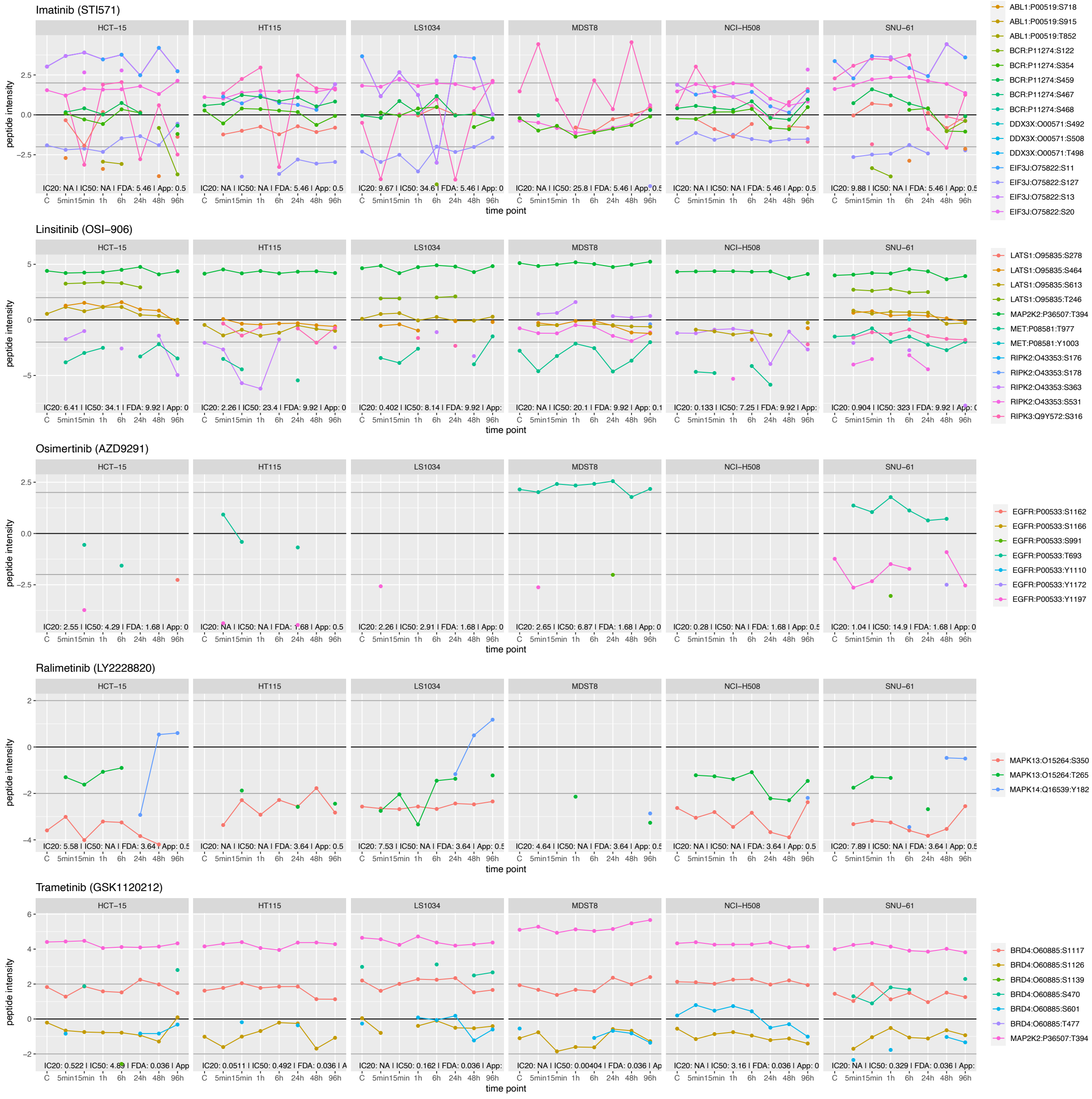


**Supplemental Figure 15. Primary drug target phosphosite abundance dynamics following perturbation.** Time dependent phosphosite abundance of the top 5 established primary targets of six drugs in each cell line. Source data are provided as a Source Data file.

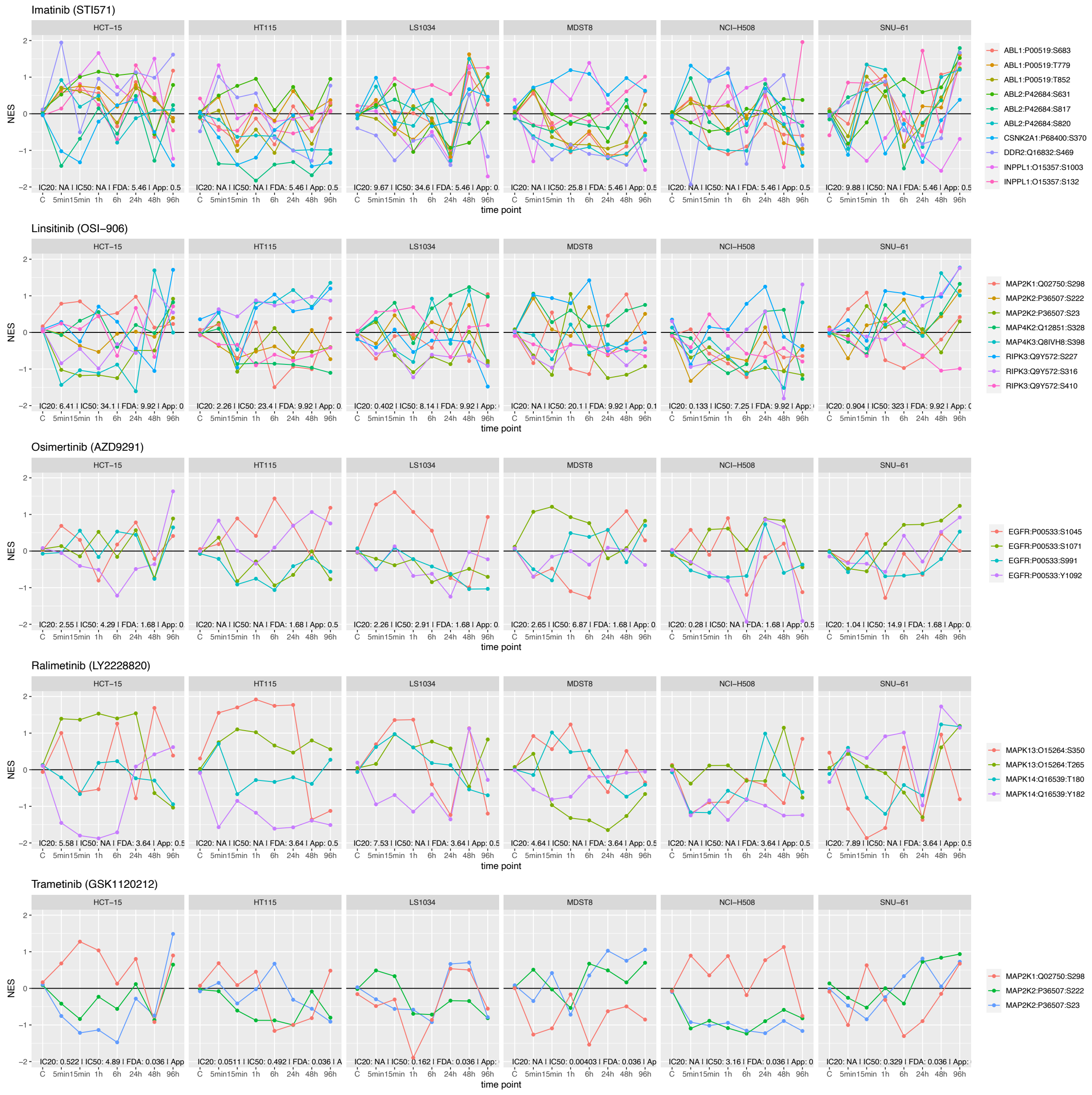


**Supplemental Figure 16. Primary drug target phosphosite-level VESPA activity dynamics following perturbation.** Time dependent activity of the top 5 established primary targets of six drugs in each cell line, as assessed by phosphosite-level VESPA analysis. Source data are provided as a Source Data file.

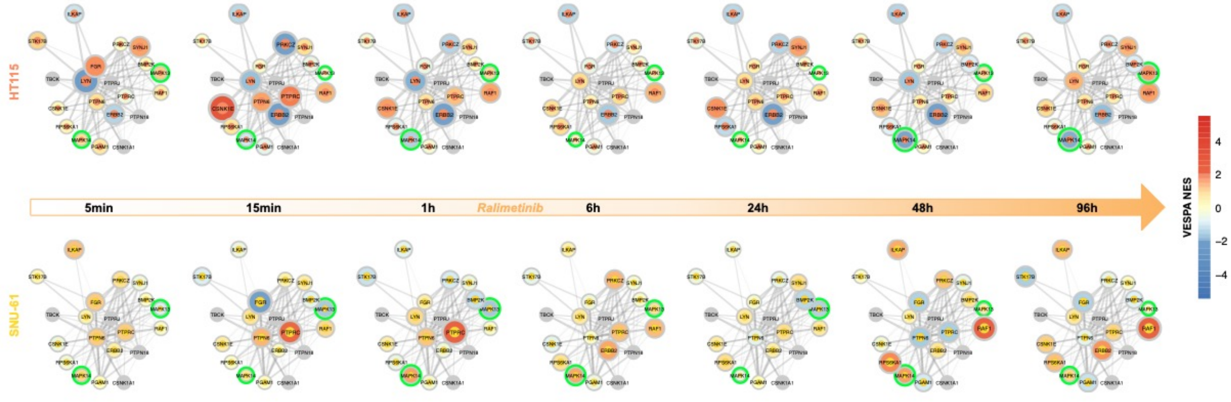


**Supplemental Figure 17. Context-specific, time-dependent signaling pathway rewiring by the MAPK inhibitor ralimetinib.** Time-dependent network dysregulation and drug mechanism of action (MoA) of ralimetinib in the HT115 and SNU-61 cell lines. Nodes indicate the most affected regulators with inner circle colors indicating cell line type and outer circle color and size indicating VESPA activity. Edges indicate dysregulated interactions (directionality not shown) between KP-enzymes (Methods), with line thickness proportional to statistical significance. Proteins highlighted by a green halo indicate established primary and off targets. Source data are provided as a Source Data file.

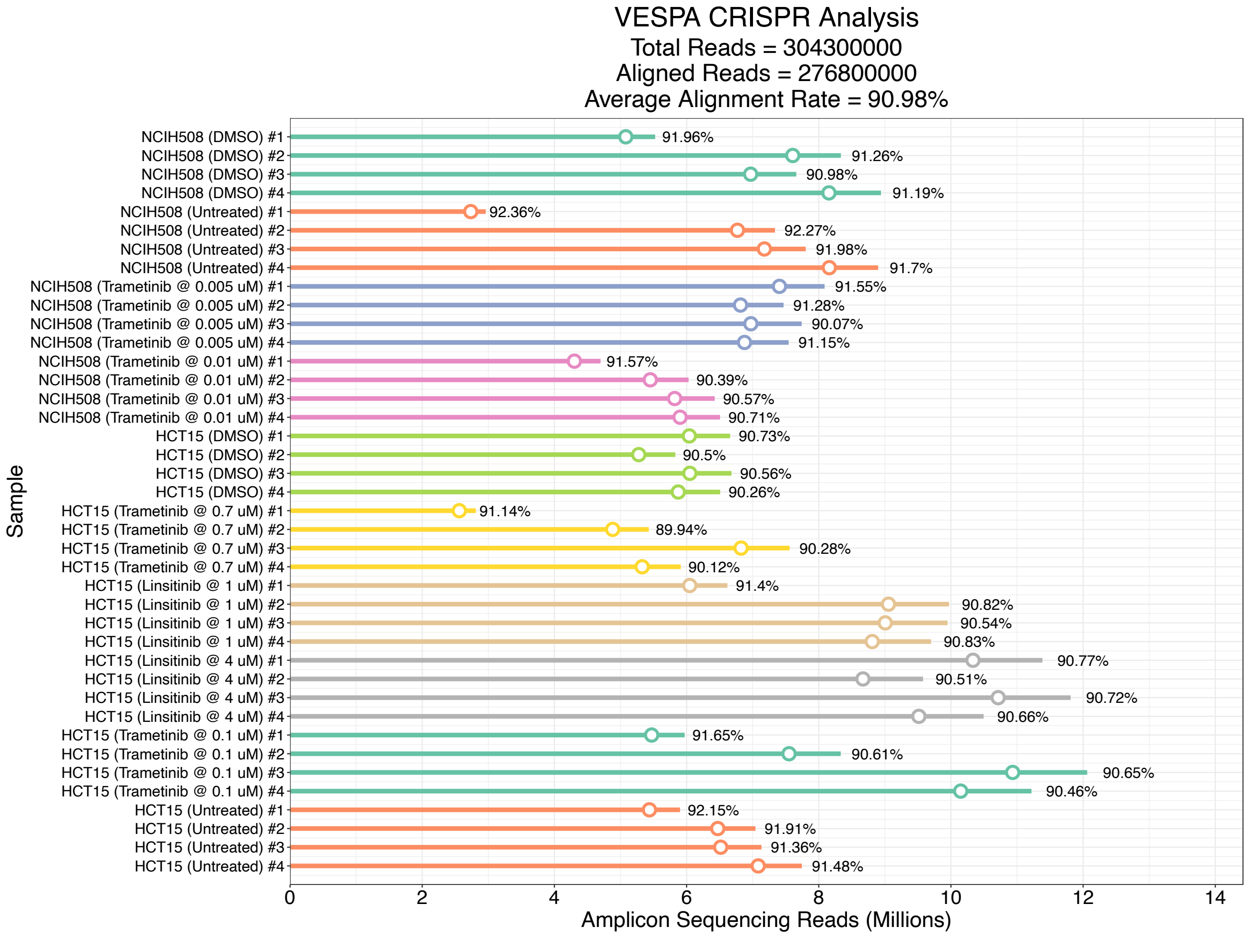


**Supplemental Figure 18. CRISPRko quality control.** The sgRNA alignment rate is shown for each experiment. Source data are provided as a Source Data file.

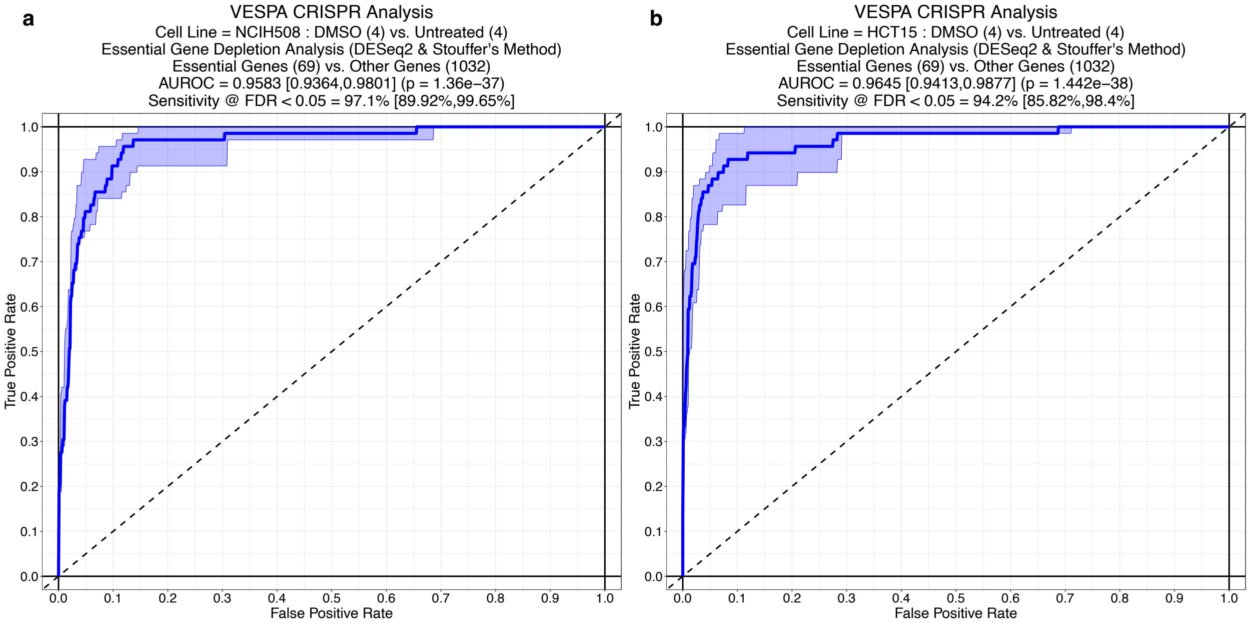


**Supplemental Figure 19. Receiver-operating-characteristics for the recovery of known essential genes by CRISPRko assay.** gRNA targeting essential genes were highly depleted in samples representing the last *vs.* the first time point (T0) of DMSO-treated cells in both **a** NCI-H508 and **b** HCT-15 cells (Methods). Source data are provided as a Source Data file.

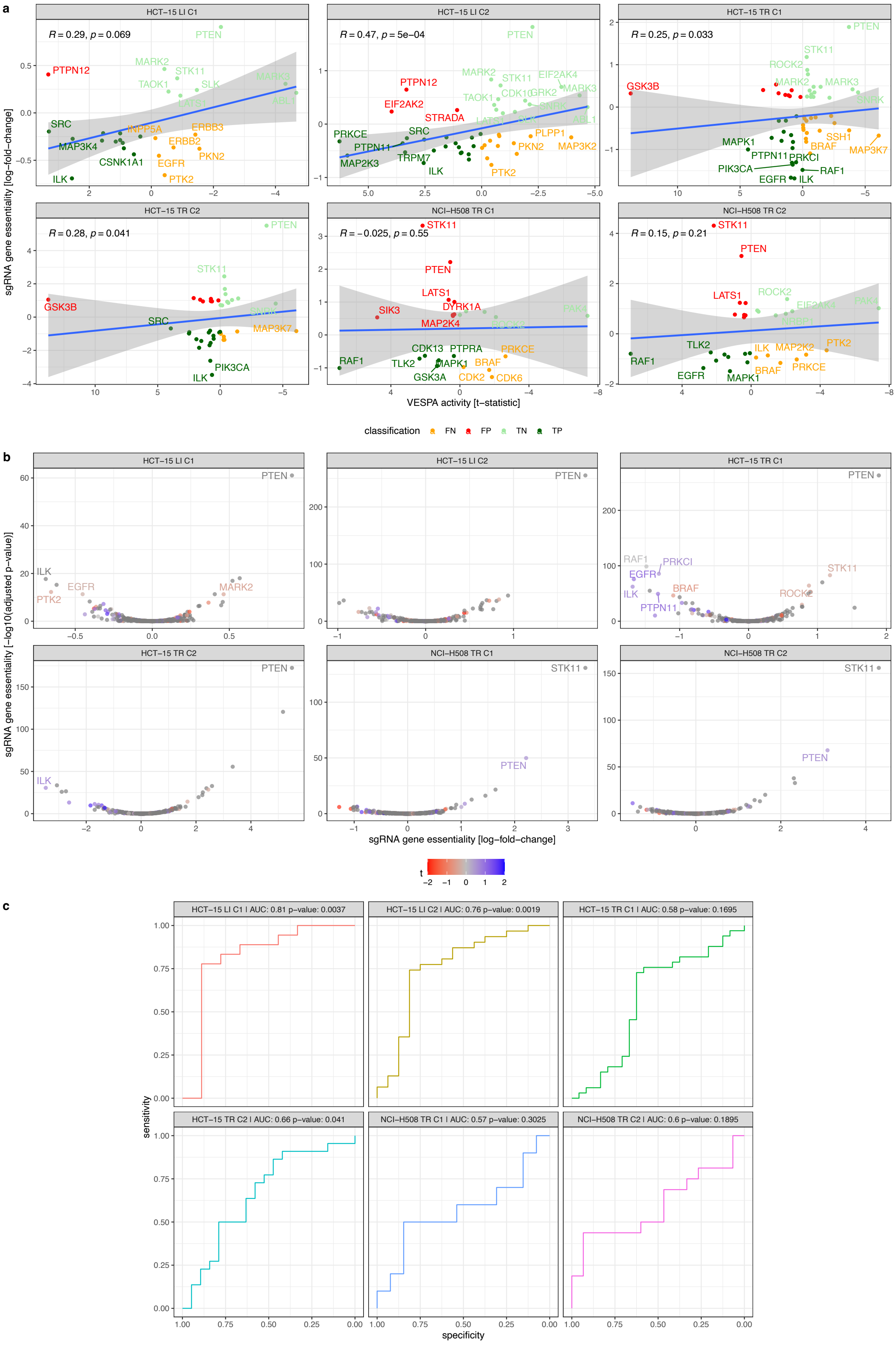
a
HCT−15 LI C1
R = 0.29, p = 0.069
HCT−15 LI C2
R = 0.47, p = 5e−04
HCT−15 TR C1
R = 0.25, p = 0.033
HCT−15 TR C2
R = 0.28, p = 0.041
NCI−H508 TR C1
R = −0.025, p = 0.55
NCI−H508 TR C2
R = 0.15, p = 0.21
sgRNA gene essentiality [log−fold−change]
VESPA activity [t−statistic]
classification FN FP TN TP
b
HCT−15 LI C1
HCT−15 LI C2
HCT−15 TR C1
HCT−15 TR C2
NCI−H508 TR C1
NCI−H508 TR C2
sgRNA gene essentiality [−log10(adjusted p−value)]
sgRNA gene essentiality [log−fold−change]
t
c
HCT−15 LI C1 | AUC: 0.81 p−value: 0.0037
HCT−15 LI C2 | AUC: 0.76 p−value: 0.0019
HCT−15 TR C1 | AUC: 0.58 p−value: 0.1695
HCT−15 TR C2 | AUC: 0.66 p−value: 0.041
NCI−H508 TR C1 | AUC: 0.57 p−value: 0.3025
NCI−H508 TR C2 | AUC: 0.6 p−value: 0.1895
sensitivity
specificity

**Supplemental Figure 20. CRISPRko-induced sensitization by proteins predicted to mediate drug resistance.** Plots showing **a** Correlation (statistics were computed using a one-tailed Spearman correlation test), **b** Volcano plot, and **c** enrichment of predicted KP-enzymes in proteins validated by CRISPRko assays is shown using a receiver operating characteristics (ROC), area under the curve (AUC), and statistical significance (one-tailed Mann-Whitney-U test). Source data are provided as a Source Data file.

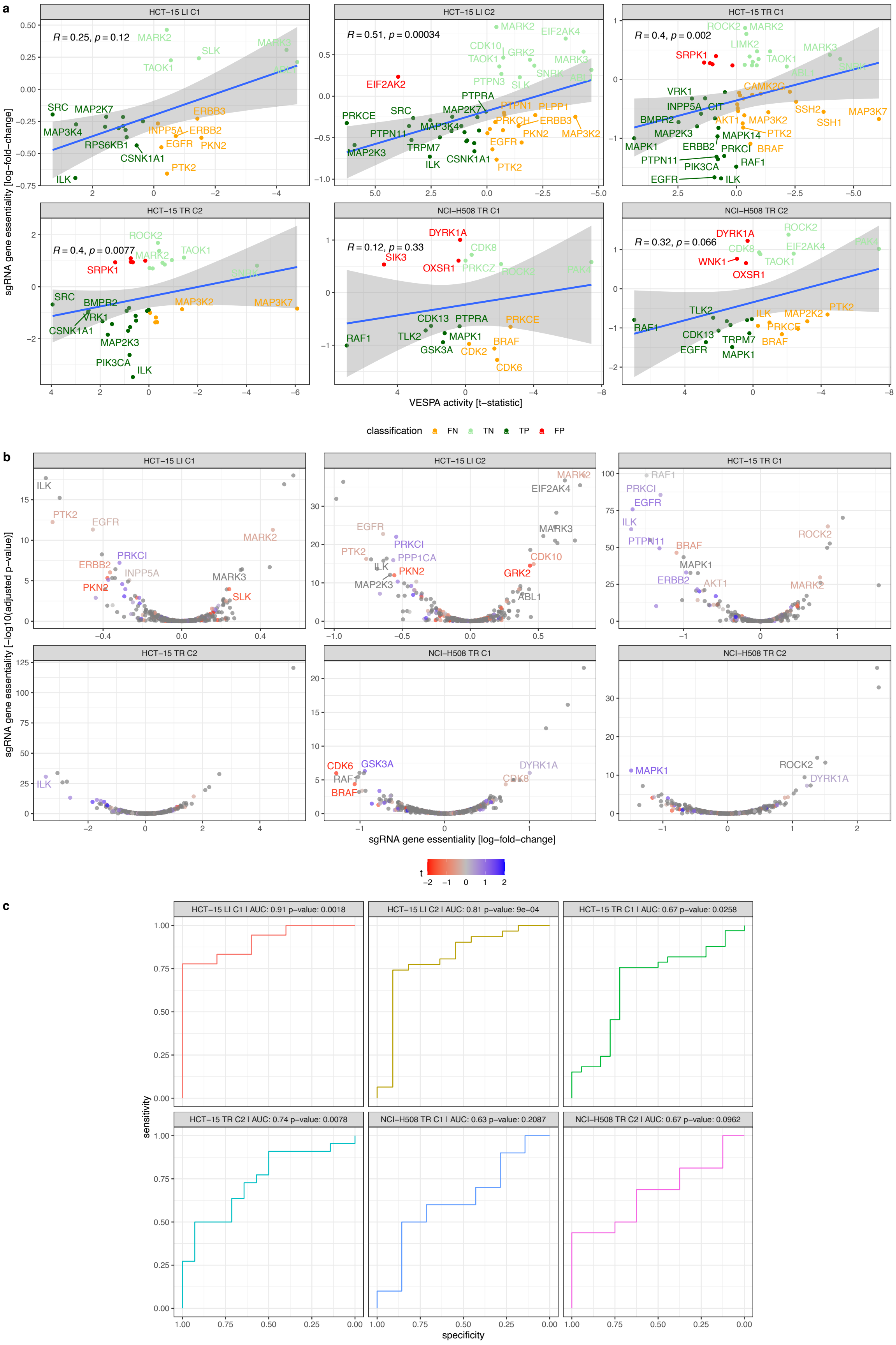

a
HCT−15 LI C1
R = 0.25, p = 0.12
HCT−15 LI C2
R = 0.51, p = 0.00034
HCT−15 TR C1
R = 0.4, p = 0.002
HCT−15 TR C2
R = 0.4, p = 0.0077
NCI−H508 TR C1
R = 0.12, p = 0.33
NCI−H508 TR C2
R = 0.32, p = 0.066
sgRNA gene essentiality [log−fold−change]
VESPA activity [t−statistic]
classification FN TN TP FP
b
HCT−15 LI C1
HCT−15 LI C2
HCT−15 TR C1
HCT−15 TR C2
NCI−H508 TR C1
NCI−H508 TR C2
sgRNA gene essentiality [−log10(adjusted p−value)]
sgRNA gene essentiality [log−fold−change]
t
c
HCT−15 LI C1 | AUC: 0.91 p−value: 0.0018
HCT−15 LI C2 | AUC: 0.81 p−value: 9e−04
HCT−15 TR C1 | AUC: 0.67 p−value: 0.0258
HCT−15 TR C2 | AUC: 0.74 p−value: 0.0078
NCI−H508 TR C1 | AUC: 0.63 p−value: 0.2087
NCI−H508 TR C2 | AUC: 0.67 p−value: 0.0962
sensitivity
specificity

**Supplemental Figure 21. CRISPRko-induced sensitization by proteins predicted to mediate drug resistance (tumor suppressor genes excluded).** Same as Supplemental Figure 20 but excluding tumor suppressor genes. Plots showing **a** Correlation (statistics were computed using a one-tailed Spearman correlation test), **b** Volcano plot, and **c** enrichment of predicted KP-enzymes in proteins validated by CRISPRko assays is shown using a receiver operating characteristics (ROC), area under the curve (AUC), and statistical significance (one-tailed Mann-Whitney-U test). Source data are provided as a Source Data file.

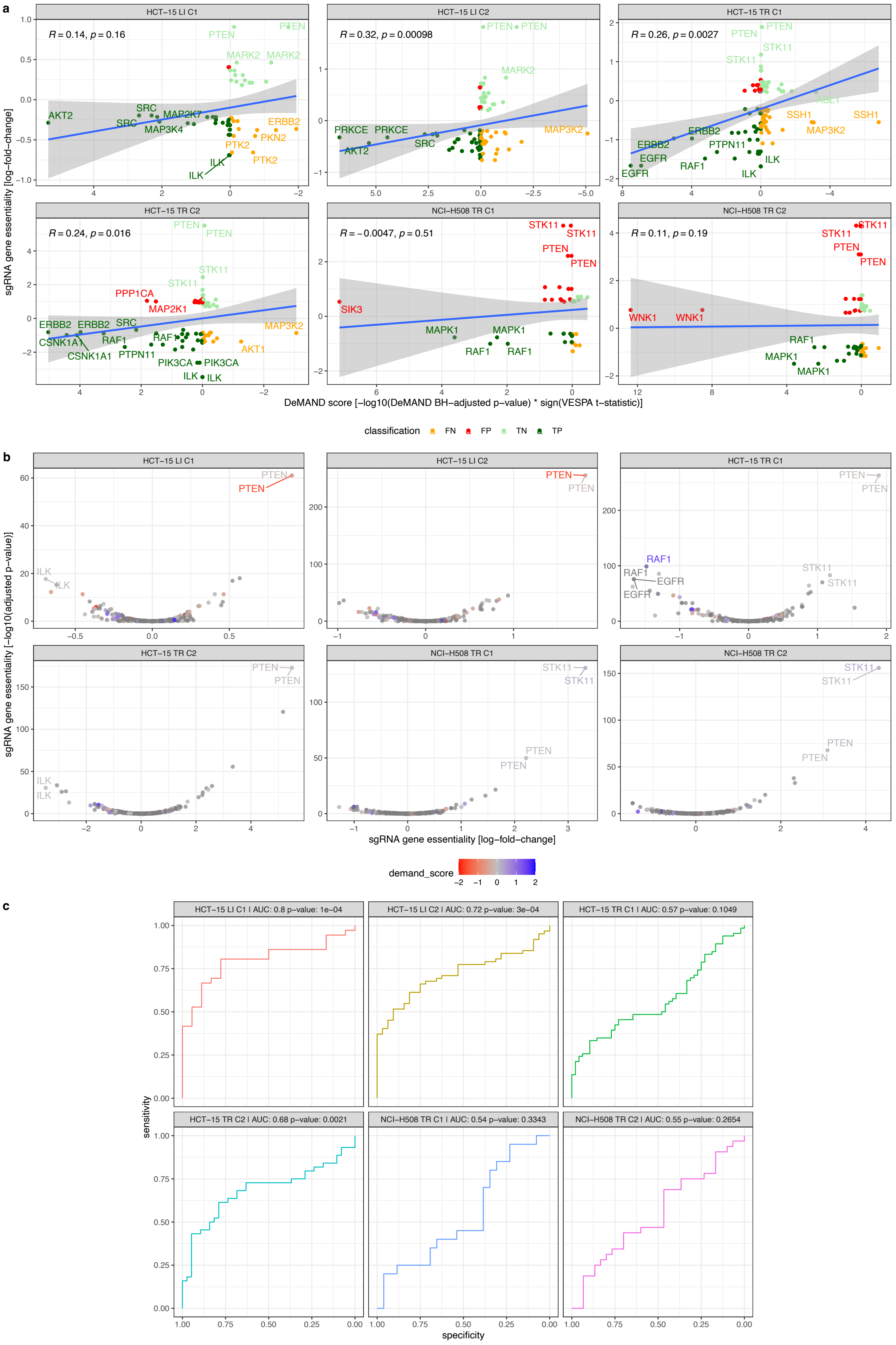
a
HCT−15 LI C1
R = 0.14, p = 0.16
HCT−15 LI C2
R = 0.32, p = 0.00098
HCT−15 TR C1
R = 0.26, p = 0.0027
HCT−15 TR C2
R = 0.24, p = 0.016
NCI−H508 TR C1
R = −0.0047, p = 0.51
NCI−H508 TR C2
R = 0.11, p = 0.19
sgRNA gene essentiality [log−fold−change]
DeMAND score [−log10(DeMAND BH−adjusted p−value) * sign(VESPA t−statistic)]
classification FN FP TN TP
b
HCT−15 LI C1
HCT−15 LI C2
HCT−15 TR C1
HCT−15 TR C2
NCI−H508 TR C1
NCI−H508 TR C2
sgRNA gene essentiality [−log10(adjusted p−value)]
sgRNA gene essentiality [log−fold−change]
demand_score
c
HCT−15 LI C1 | AUC: 0.8 p-value: 1e−04
HCT−15 LI C2 | AUC: 0.72 p-value: 3e−04
HCT−15 TR C1 | AUC: 0.57 p-value: 0.1049
HCT−15 TR C2 | AUC: 0.68 p-value: 0.0021
NCI−H508 TR C1 | AUC: 0.54 p-value: 0.3343
NCI−H508 TR C2 | AUC: 0.55 p-value: 0.2654
sensitivity
specificity

**Supplemental Figure 22. CRISPRko-induced sensitization by proteins predicted to mediate drug resistance (VESPA & DeMAND predictions).** Same as Supplemental Figure 20 but with predictions based on the integration of VESPA and DeMAND. Plots showing **a** Correlation (statistics were computed using a one-tailed Spearman correlation test), **b** Volcano plot, and **c** enrichment of predicted KP-enzymes in proteins validated by CRISPRko assays is shown using a receiver operating characteristics (ROC), area under the curve (AUC), and statistical significance (one-tailed Mann-Whitney-U test). Source data are provided as a Source Data file.

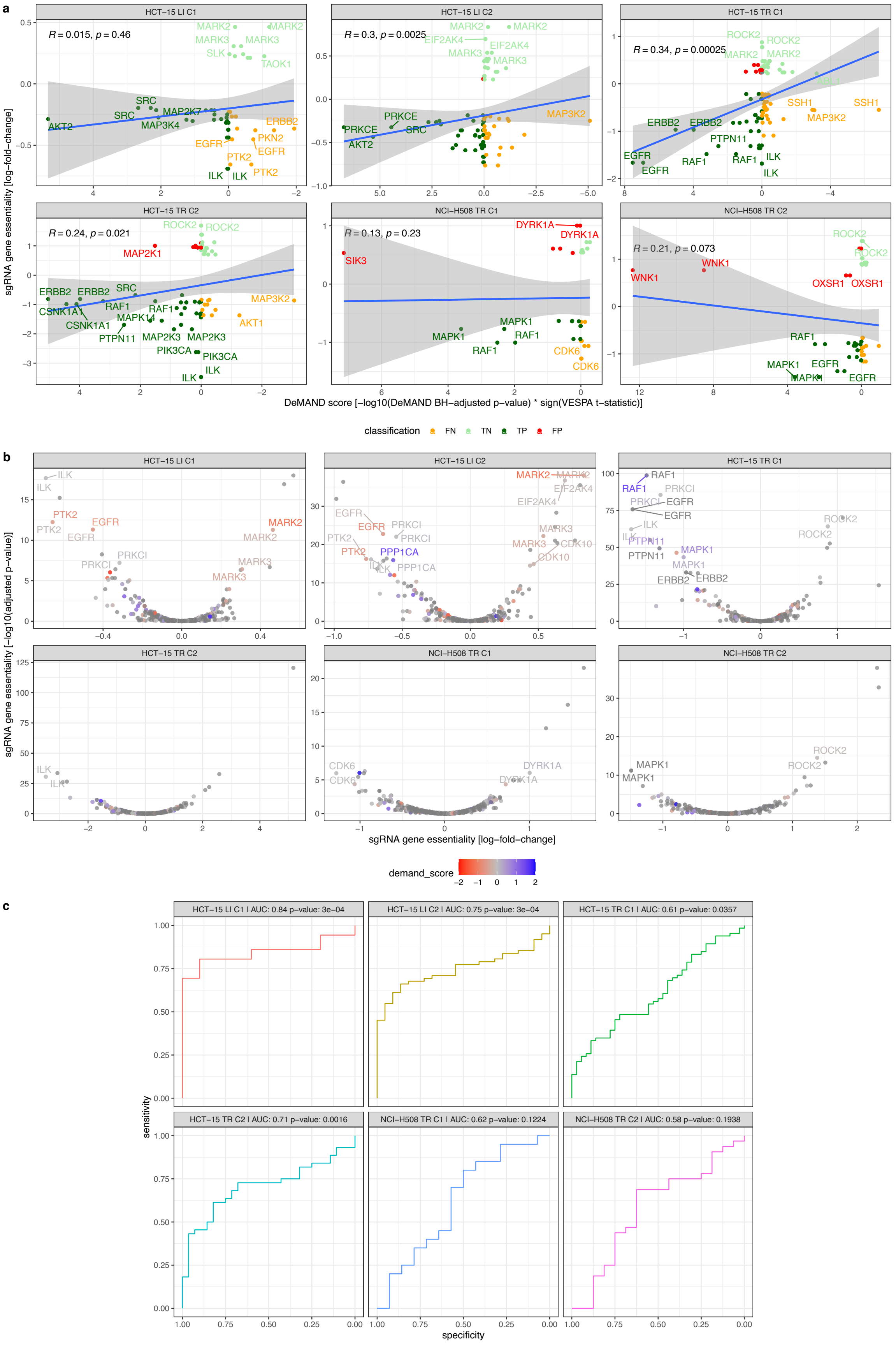
a
HCT−15 LI C1
R = 0.015, p = 0.46
HCT−15 LI C2
R = 0.3, p = 0.0025
HCT−15 TR C1
R = 0.34, p = 0.00025
HCT−15 TR C2
R = 0.24, p = 0.021
NCI−H508 TR C1
R = 0.13, p = 0.23
NCI−H508 TR C2
R = 0.21, p = 0.073
sgRNA gene essentiality [log−fold−change]
DeMAND score [−log10(DeMAND BH−adjusted p−value) * sign(VESPA t−statistic)]
classification FN TN TP FP
b
HCT−15 LI C1
HCT−15 LI C2
HCT−15 TR C1
HCT−15 TR C2
NCI−H508 TR C1
NCI−H508 TR C2
sgRNA gene essentiality [−log10(adjusted p−value)]
sgRNA gene essentiality [log−fold−change]
demand_score
c
HCT−15 LI C1 | AUC: 0.84 p-value: 3e-04
HCT−15 LI C2 | AUC: 0.75 p-value: 3e-04
HCT−15 TR C1 | AUC: 0.61 p-value: 0.0357
HCT−15 TR C2 | AUC: 0.71 p-value: 0.0016
NCI−H508 TR C1 | AUC: 0.62 p-value: 0.1224
NCI−H508 TR C2 | AUC: 0.58 p-value: 0.1938
sensitivity
specificity

**Supplemental Figure 23. CRISPRko-induced sensitization by proteins predicted to mediate drug resistance (VESPA & DeMAND predictions; tumor suppressor genes excluded).** Same as Supplemental Figure 22 but excluding tumor suppressor genes. Plots showing **a** Correlation (statistics were computed using a one-tailed Spearman correlation test), **b** Volcano plot, and **c** enrichment of predicted KP-enzymes in proteins validated by CRISPRko assays is shown using a receiver operating characteristics (ROC), area under the curve (AUC), and statistical significance (one-tailed Mann-Whitney-U test). Source data are provided as a Source Data file.

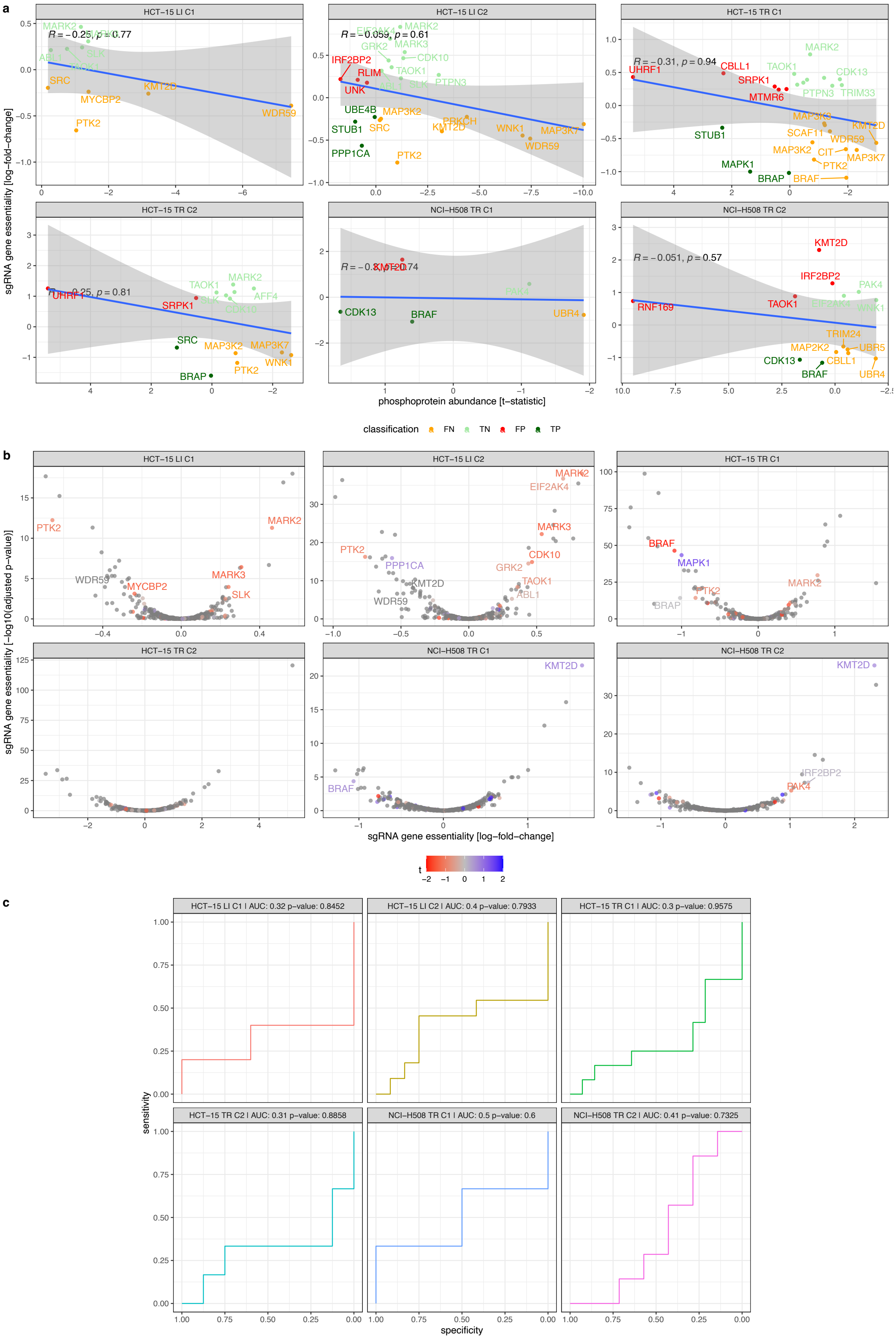
a
HCT−15 LI C1
HCT−15 LI C2
HCT−15 TR C1
HCT−15 TR C2
NCI−H508 TR C1
NCI−H508 TR C2
R = −0.25, p = 0.77
R = −0.059, p = 0.61
R = −0.31, p = 0.94
R = −0.051, p = 0.57
sgRNA gene essentiality [log−fold−change]
phosphoprotein abundance [t−statistic]
classification FN TN FP TP
b
sgRNA gene essentiality [−log10(adjusted p−value)]
sgRNA gene essentiality [log−fold−change]
t
c
HCT−15 LI C1 | AUC: 0.32 p−value: 0.8452
HCT−15 LI C2 | AUC: 0.4 p−value: 0.7933
HCT−15 TR C1 | AUC: 0.3 p−value: 0.9575
HCT−15 TR C2 | AUC: 0.31 p−value: 0.8858
NCI−H508 TR C1 | AUC: 0.5 p−value: 0.6
NCI−H508 TR C2 | AUC: 0.41 p−value: 0.7325
sensitivity
specificity

**Supplemental Figure 24. CRISPRko-induced sensitization by proteins predicted to mediate drug resistance (differential phosphosite abundance).** Same as Supplemental Figure 20 but with predictions based on differential phosphosite abundance analysis. Plots showing **a** Correlation (statistics were computed using a one-tailed Spearman correlation test), **b** Volcano plot, and **c** enrichment of predicted KP-enzymes in proteins validated by CRISPRko assays is shown using a receiver operating characteristics (ROC), area under the curve (AUC), and statistical significance (one-tailed Mann-Whitney-U test). Source data are provided as a Source Data file.

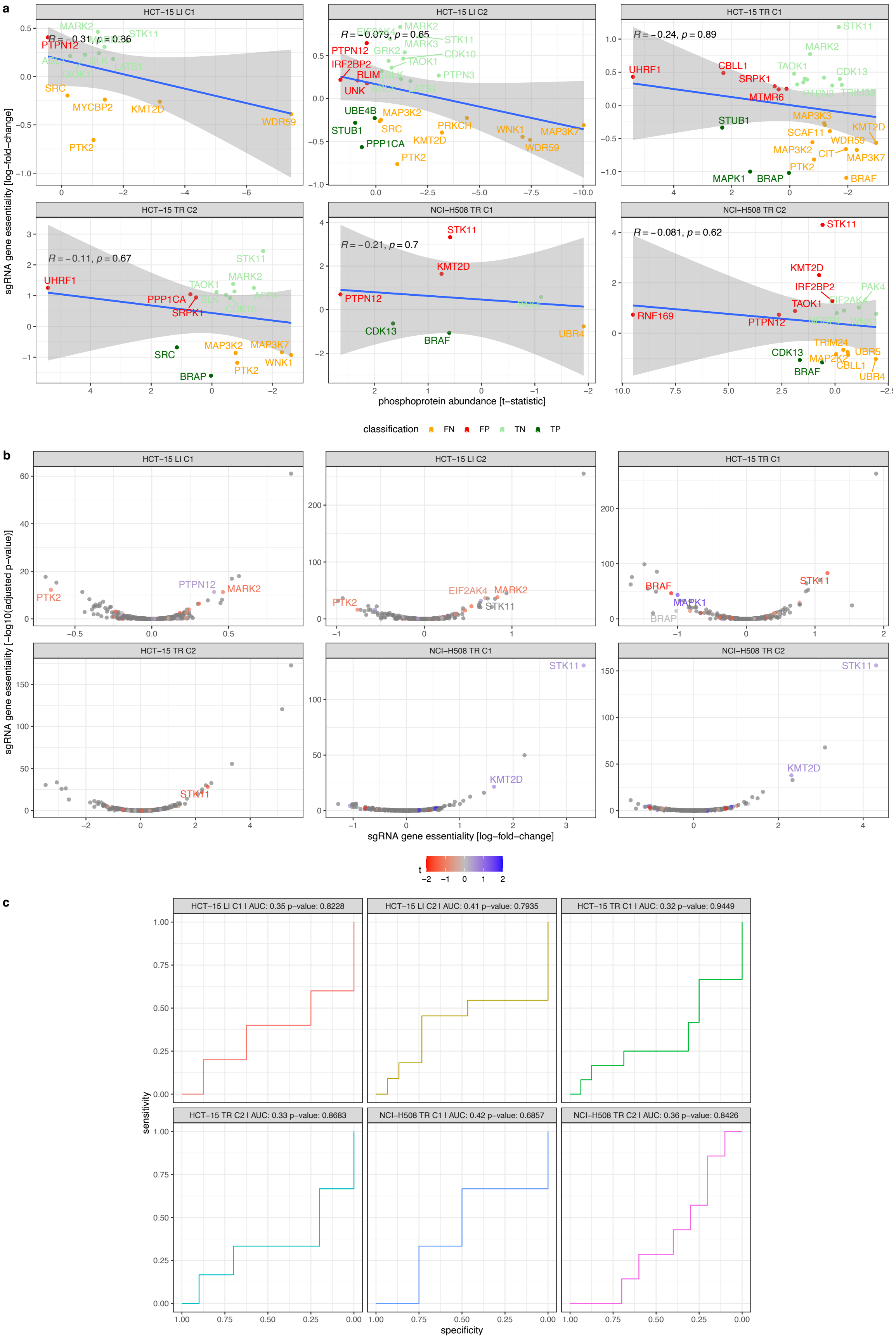
a
HCT−15 LI C1
HCT−15 LI C2
HCT−15 TR C1
HCT−15 TR C2
NCI−H508 TR C1
NCI−H508 TR C2
R = −0.31, p = 0.36
R = −0.079, p = 0.65
R = −0.24, p = 0.89
R = −0.11, p = 0.67
R = −0.21, p = 0.7
R = −0.081, p = 0.62
sgRNA gene essentiality [log−fold−change]
phosphoprotein abundance [t−statistic]
classification FN FP TN TP
b
HCT−15 LI C1
HCT−15 LI C2
HCT−15 TR C1
HCT−15 TR C2
NCI−H508 TR C1
NCI−H508 TR C2
sgRNA gene essentiality [−log10(adjusted p−value)]
sgRNA gene essentiality [log−fold−change]
t
c
HCT−15 LI C1 | AUC: 0.35 p−value: 0.8228
HCT−15 LI C2 | AUC: 0.41 p−value: 0.7935
HCT−15 TR C1 | AUC: 0.32 p−value: 0.9449
HCT−15 TR C2 | AUC: 0.33 p−value: 0.8683
NCI−H508 TR C1 | AUC: 0.42 p−value: 0.6857
NCI−H508 TR C2 | AUC: 0.36 p−value: 0.8426
sensitivity
specificity

**Supplemental Figure 25. CRISPRko-induced sensitization by proteins predicted to mediate drug resistance (differential phosphosite abundance; tumor suppressor genes excluded).** Same as Supplemental Figure 24 but excluding tumor suppressor genes. Plots showing **a** Correlation (statistics were computed using a one-tailed Spearman correlation test), **b** Volcano plot, and **c** enrichment of predicted KP-enzymes in proteins validated by CRISPRko assays is shown using a receiver operating characteristics (ROC), area under the curve (AUC), and statistical significance (one-tailed Mann-Whitney-U test). Source data are provided as a Source Data file.

**Supplementary Table 1.** IC20 screening data

| Category | Parameter | Description |
|---|---|---|
| Assay | Type of assay | Cell-Based $IC_{20}$ determination |
| | Target | N/A |
| | Primary measurement | ATP quantification |
| | Key reagents | Cell-TiterGlo Promega Corp |
| | Assay protocol | See $IC_{20}$ Determination in Methods |
| | Additional comments | |
| Library | Library size | 7 drugs |
| | Library composition | Known Bioactives |
| | Source | SelectChem |
| | Additional comments | |
| Screen | Format | 384-Well |
| | Concentration(s) tested | 10μM to 25nM |
| | Plate controls | DMSO positive control, Thimerosal positive control |
| | Reagent/ compound dispensing system | Beckman Coulter Echo |
| | Detection instrument and software | Revity EnVision |
| | Assay validation/QC | Z' for plate, $R^2$ for curves |
| | Correction factors | N/A |
| | Normalization | Normalized Viability calculated from controls |
| | Additional comments | |
| Post-HTS analysis | Hit criteria | Successful fit of four-parameter sigmoid models |
| | Hit rate | 23 / 36: 63.9% |
| | Additional assay(s) | N/A |
| | Confirmation of hit purity and structure | N/A |
| | Additional comments | |

**Supplementary Table 2.** Pooled CRISPR-ko screens

| Category | Parameter | Description |
|---|---|---|
| Assay | Type of assay | *in vitro* screen using HCT-15 and NCI-H508 cell lines |
| | Target | All kinases, phosphatases and E3-ligases (see Supplemental Table 16) |
| | Primary measurement | Amplicon seq NGS |
| | Key reagents | Trametinib, Linsitinib, DMSO |
| | Assay protocol | Please see the CRISPRko-section in the materials and methods |
| | Additional comments | |
| Library | Library size | 1101 genes, all targeted with 4 sgRNAs |
| | Library composition | sgRNAs targeting all kinases, phosphatases and E3-ligases |
| | Source | The library was designed by using CRISPick |
| | Additional comments | Please see the CRISPRko-section in the materials and methods |
| Screen | Format | 15cm plate format |
| | Concentration(s) tested | Trametinib:<br>HCT-15: C1: 0.1 µM, C2: 0.7 µM<br>NCI-H508: C1: 0.005 *µ*M, C2: 0.01 *µ*M<br><br>Linsitinib:<br>HCT-15: C1: 1.0 *µ*M, C2: 4.0 *µ*M |
| | Plate controls | DMSO: 0.15% for all screens |
| | Reagent/ compound dispensing system | Manual cell culturing using 15cm plate format |
| | Detection instrument and software | Illumina PE150 Sequencing (NovaSeq) |
| | Assay validation/QC | Average alignment rate (ShortRead): 90.98%<br>Essential gene recovery: AUROC 0.96 (Supplemental Figure 19) |
| | Correction factors | N/A |
| | Normalization | DESeq2 (see Methods: Identification of essential genes using DESeq2) |
| | Additional comments | Please see the CRISPRko-section in the materials and methods |
| Post-HTS analysis | Hit criteria | N/A |
| | Hit rate | N/A |
| | Additional assay(s) | N/A |
| | Confirmation of hit purity and structure | N/A |
| | Additional comments | |
