# Supplementary material for: Network-based elucidation of colon cancer drug resistance mechanisms by phosphoproteomic time-series analysis
Source: Nat Commun. 2024 May 9;15:3909. doi: 10.1038/s41467-024-47957-3 (PMC11082183; doi:10.1038/s41467-024-47957-3)
Supplement: Supplementary file 3 — Description of Additional Supplementary Files [file 41467_2024_47957_MOESM3_ESM.pdf]

# Description of Additional Supplementary Files

**Supplemental Data 1. VESPA CRC signalons.** Using the CPTAC-S045, CPTAC-S045N and U54-NET datasets, dVESPA was used to generate CRC-specific signalons. This archive contains the corresponding R data structures that can be readily used with mVESPA.

**Supplemental Data 2. mVESPA Benchmarking: AUROC performance characteristics.** For different comparisons, AUROC values have been computed. This table provides the values for all combinations.

**Supplemental Data 3. mVESPA Benchmarking: Statistical significance of relative performance comparison.** For different comparisons, the statistical significance of comparison (one-tailed Wilcox' test) has been computed. This table provides the values for all combinations.

**Supplemental Data 4. Cell Line Selection for CRC Analysis: VESPA analysis results.** VESPA was used to guide the CRC cell line selection. This table provides the main VESPA NES values that underly the corresponding analyses.

**Supplemental Data 5. Cell Line Selection for CRC Analysis: VESPA feature importance.** VESPA was used to guide the CRC cell line selection. In a second step, the VESPA NES values were used for feature selection with the goal to identify the most characteristic K/P-enzymes for each VESPA cluster (VC). This table provides the Random Forest feature importance values for each VC and K/P-enzyme.

**Supplemental Data 6. Cell Line Selection for CRC Analysis: CMS feature importance.** VESPA was used to guide the CRC cell line selection. In a second step, the VESPA NES values were used for feature selection with the goal to identify the most characteristic K/P-enzymes for each CMS group. This table provides the Random Forest feature importance values for each CMS group and K/P-enzyme.

**Supplemental Data 7. Cell Line Selection for CRC Analysis: GSEA analysis results.** VESPA was used to guide the CRC cell line selection. In a second step, the VESPA NES values were used for gene set enrichment analysis (GSEA) to identify the involved pathways. This table provides the GSEA NES values for all comparisons.

**Supplemental Data 8. Preprocessed MS data for this publication; compatible with VESPA.** This table provides the preprocessed phosphoproteomic and proteomic profiles generated for this study. Several files are supplied that provide drug perturbation profiles (U54DP_COAD_phospho.rds), baseline profiles

(U54BL_COAD_proteo.rds, U54BL_COAD_phospho.rds) and the combined dataset (U54_COAD_phospho.rds). Profiles are provided in long list format, directly compatible with mVESPA.

**Supplemental Data 9. Generation of Drug Perturbation Profiles: Integrated-level VESPA results.** mVESPA was used to process drug perturbation times series phosphoproteomic data with the corresponding CRC signalons. This table provides the integrated-level VESPA NES values.

**Supplemental Data 10. Generation of Drug Perturbation Profiles: Phosphostate-level VESPA results.** mVESPA was used to process drug perturbation times series phosphoproteomic data with the corresponding CRC signalons. This table provides the phosphostate-level VESPA NES values.

**Supplemental Data 11. Generation of Drug Perturbation Profiles: Activity-level VESPA results.** mVESPA was used to process drug perturbation times series phosphoproteomic data with the corresponding CRC signalons. This table provides the activity-level VESPA NES values.

**Supplemental Data 12. Generation of Drug Perturbation Profiles: GSEA results.** mVESPA was used to process drug perturbation times series phosphoproteomic data with the corresponding CRC signalons. In a second step, the VESPA NES values were used for gene set enrichment analysis (GSEA) to identify the involved pathways. This table provides the GSEA NES values.

**Supplemental Data 13. Generation of Drug Perturbation Profiles: Phosphosite-level VESPA results.** mVESPA was used to process drug perturbation times series phosphoproteomic data with the corresponding CRC signalons. This table provides the phosphosite-level VESPA NES values.

**Supplemental Data 14. Context-specific Signaling Network Adaptation and Rewiring: DeMAND results on Node level.** Using the inferred VESPA K/P-enzyme activities, DeMAND was used to elucidate signaling network adaptation and rewiring. This table provides the node-level DeMAND results.

**Supplemental Data 15. Context-specific Signaling Network Adaptation and Rewiring: DeMAND results on Edge level.** Using the inferred VESPA K/P-enzyme activities, DeMAND was used to elucidate signaling network adaptation and rewiring. This table provides the edge-level DeMAND results.

**Supplemental Data 16. Preprocessed DeMAND temporal network profile visualizations.** Using the inferred VESPA K/P-enzyme activities, DeMAND was used to elucidate signaling network adaptation and rewiring. This archive provides the temporal, integrated Cytoscape network visualizations in SVG format for all conditions.

**Supplemental Data 17. Cell Adaptation-mediated Drug Resistance: VESPA differential analysis results.** To identify K/P-enzymes involved in cell adaptation mediated drug resistance, differential analysis of VESPA NES values was conducted between the late *vs.* early time points. This table provides the differential testing analysis results for all conditions separately.

**Supplemental Data 18. Cell Adaptation-mediated Drug Resistance: Aggregated VESPA differential analysis results.** To identify K/P-enzymes involved in cell adaptation mediated drug resistance, differential analysis of VESPA NES values was conducted between the late *vs.* early time points. This table provides the differential testing analysis results, aggregated across all conditions.

**Supplemental Data 19. Experimental Validation by CRISPR/Cas9-mediated Silencing: Guide RNA sequences for target genes.** This table provides guide RNA sequences for the target genes of the CRISPR-ko screen.

**Supplemental Data 20. Experimental Validation by CRISPR/Cas9-mediated Silencing: DESeq2 analysis results.** CRISPR-ko screening data was analyzed using DEseq2. This table provides the results of the differential analysis.
