# Supplementary material for: Network-based elucidation of colon cancer drug resistance mechanisms by phosphoproteomic time-series analysis
Source: Nat Commun. 2024 May 9;15:3909. doi: 10.1038/s41467-024-47957-3 (PMC11082183; doi:10.1038/s41467-024-47957-3)
Supplement: Supplementary file 24 — Reporting Summary [file 41467_2024_47957_MOESM24_ESM.pdf]

Corresponding author(s): Andrea Califano, Yansheng Liu

Last updated by author(s): 04/03/2024

Reporting Summary

Nature Portfolio wishes to improve the reproducibility of the work that we publish. This form provides structure for consistency and transparency in reporting. For further information on Nature Portfolio policies, see our Editorial Policies and the Editorial Policy Checklist.

Statistics

For all statistical analyses, confirm that the following items are present in the figure legend, table legend, main text, or Methods section.

n/a	Confirmed	
☐	☒	The exact sample size (*n*) for each experimental group/condition, given as a discrete number and unit of measurement
☐	☒	A statement on whether measurements were taken from distinct samples or whether the same sample was measured repeatedly
☐	☒	The statistical test(s) used AND whether they are one- or two-sided<br>*Only common tests should be described solely by name; describe more complex techniques in the Methods section.*
☒	☐	A description of all covariates tested
☐	☒	A description of any assumptions or corrections, such as tests of normality and adjustment for multiple comparisons
☐	☒	A full description of the statistical parameters including central tendency (e.g. means) or other basic estimates (e.g. regression coefficient) AND variation (e.g. standard deviation) or associated estimates of uncertainty (e.g. confidence intervals)
☐	☒	For null hypothesis testing, the test statistic (e.g. *F*, *t*, *r*) with confidence intervals, effect sizes, degrees of freedom and *P* value noted<br>*Give P values as exact values whenever suitable.*
☒	☐	For Bayesian analysis, information on the choice of priors and Markov chain Monte Carlo settings
☒	☐	For hierarchical and complex designs, identification of the appropriate level for tests and full reporting of outcomes
☐	☒	Estimates of effect sizes (e.g. Cohen's *d*, Pearson's *r*), indicating how they were calculated

Our web collection on statistics for biologists contains articles on many of the points above.

Software and code

Policy information about availability of computer code

Data collection

Vendor-provided software was used for proteomic data acquisition. Full details and version information can be obtained from instrument raw data provided on the public repositories.

Data analysis

Only the open source software and versions listed in the Methods section was used for all analysis:

Mass spectrometry data analysis:
All raw data files were processed and converted to mzXML by ProteoWizard (version 3.0), enabling centroiding (using the vendor-provided algorithm) on MS1 and MS2 levels. For peptide identification and quantification, an integrated Snakemake workflow consisting of DIA-Umpire (version 2.1.6), MSFragger (version 2.3.0), the Trans-Proteomic Pipeline (PeptideProphet, PTMProphet, iProphet, versio Kn 5.2.0), EasyPQP (version 0.1.6), OpenSWATH (OpenMS, version 2.5.0), PyProphet (version 2.1.4) and TRIC (msproteomicstools, version 0.11.0) was used. For quantitative protein abundance inference, the R-package “iq“ (version 1.9), implementing the MaxLFQ algorithm for DIA-based datasets, was used with default parameters.

Data processing & statistical analysis:
For all data analysis steps, “viper” (version 1.22.0), “vespa” (version 1.0.2), “vespa.db” (version 1.0.2) and “vespa.aracne” (version 2.2) were used. “vespa.net” (version 1.0.2) was executed using the corresponding Docker images of the algorithms converted to Singularity images. All software tools are available from the corresponding repositories as referred below. To compute dMI, “vespa.aracne” was extended to support depletion (Git branch “depletion_support”; revision 470944f).

Receiver-Operating-Characteristics (ROC) curves were generated using the pROC R-package (version 1.17.0.1) and default parameters. P-values for ROC curve comparisons were also computed using pROC by DeLong’s test and using default parameters. Precision-recall curves

(PRC) and corresponding metrics were computed using the PRROC R-package (version 1.3.1). Statistical comparison of the differential comparison AUC metrics were conducted using an unpaired, right tailed Wilcox' tests (R-package "stats", version 4.2.1). KSTAR (version: https://github.com/NaegleLab/KSTAR_Applications/tree/95563ddc57d39c200f06dd78a2c3672cd2d04bf2) and the provided precomputed results were used for the comparison of algorithms. GSEA was conducted by the R-package "fgsea" (version 1.14.0). Feature importance was assessed by applying the Random Forest recursive feature elimination method from the R-package "caret" (version 6.0-86). Heatmaps were generated using the "pheatmap" (version 1.0.12) R-package. Violin plots were generated using the "geom_violin" function with default parameters of the R-package "ggplot2" (version 3.4.0). The DeMAND (version 1.18.0) algorithm was used to assess context-specific wiring of signaling pathways. To visualize the interaction networks, Cytoscape (version 3.8.2) was used. For quantitative phosphoprotein abundance inference, the R-package "iq" (version 1.9), implementing the MaxLFQ algorithm105 for DIA-based datasets, was used with default parameters. Alignment of NGS with sgRNA guides was conducted using the "ShortRead" R-package (1.54.0). Differential expression analysis was conducted separately for each guide with the "DESeq2" R-package (1.36.0). ROC p-values were computed using the function "roc.area" from the R-package "validation" (version: 1.42). Correlation statistics were computed using a one-tailed Spearman correlation test (R version 4.2.1).

VESPA is available as modular platform-independent open-source software under a non-commercial usage license. VESPA consists of five different modules, which are provided as versioned source code, binaries or docker containers.
• The "vespa" R-package for signaling protein activity inference is available from GitHub (https://github.com/califano-lab/vespa) and Zenodo (https://zenodo.org/doi/10.5281/zenodo.10731059).
• The "vespa.db" R-package providing preprocessed reference networks is available from GitHub (https://github.com/califano-lab/vespa.db) and Zenodo (https://zenodo.org/doi/10.5281/zenodo.10731069).
• The "vespa.aracne" algorithm with is available from GitHub (https://github.com/califano-lab/vespa.aracne) and Zenodo (https://zenodo.org/doi/10.5281/zenodo.10731065).
• The "vespa.net" Snakemake workflow to generate context-specific signalons from one or multiple datasets is available from GitHub (https://github.com/califano-lab/vespa.net) and Zenodo (https://zenodo.org/doi/10.5281/zenodo.10731073).
• A tutorial describing the full analysis workflow with example data is available from GitHub (https://github.com/califano-lab/vespa.tutorial) and Zenodo (https://zenodo.org/doi/10.5281/zenodo.10731075).

For manuscripts utilizing custom algorithms or software that are central to the research but not yet described in published literature, software must be made available to editors and reviewers. We strongly encourage code deposition in a community repository (e.g. GitHub). See the Nature Portfolio guidelines for submitting code & software for further information.

Data

Policy information about availability of data

All manuscripts must include a data availability statement. This statement should provide the following information, where applicable:

- Accession codes, unique identifiers, or web links for publicly available datasets
- A description of any restrictions on data availability
- For clinical datasets or third party data, please ensure that the statement adheres to our policy

Original data:
The CRC mass spectrometry proteomics raw and preprocessed data generated in this study have been deposited to the ProteomeXchange Consortium via the MassIVE partner repository with the data set identifiers MSV000091204 / PXD039859 [https://doi.org/doi:10.25345/C5R20S61Q].
The CRISPRko RNA-seq raw and preprocessed data discussed in this publication have been deposited in NCBI's Gene Expression Omnibus132 and are accessible through GEO Series accession number GSE224396 [https://www.ncbi.nlm.nih.gov/geo/query/acc.cgi?acc=GSE224396].
The VESPA analysis results for selected CPTAC datasets are available from Zenodo [https://doi.org/10.5281/zenodo.8220610].
Supplemental Data 1-20 is available from Zenodo [https://doi.org/10.5281/zenodo.10925250].
Figure source data are provided with this paper.

Public data:
CCPTAC mass spectrometry proteomics raw and preprocessed data (PDC000116 and PDC000117) was obtained from Proteomic Data Commons (PDC) under the Creative Commons CC-BY 4.0 licensing terms: [https://proteomic.datacommons.cancer.gov/pdc/].

Used reference datasets and databases:
UniProtKB/Swiss-Prot Human FASTA (https://www.uniprot.org)
List of kinases (https://ftp.uniprot.org/pub/databases/uniprot/knowledgebase/complete/docs/pkinfam.txt)
List of phosphatases (https://doi.org/10.1126/scisignal.2003203)
LinkPhinder DB (https://doi.org/10.1371/journal.pcbi.1007578)
PathwayCommons (version 12; https://www.pathwaycommons.org)
HSM/P (https://doi.org/10.1038%2Fs41592-019-0687-1)
INKA (https://doi.org/10.15252%2Fmsb.20188250)
Reactome pathways (version 75; https://reactome.org)
DrugBank (https://go.drugbank.com)
ProteomicsDB (https://www.proteomicsdb.org)
STRING PPI DB (version 11; https://string-db.org)
DepMap (https://depmap.org/portal/)
TSGene 2.0 (https://bioinfo.uth.edu/TSGene/)

Research involving human participants, their data, or biological material

Policy information about studies with human participants or human data. See also policy information about sex, gender (identity/presentation), and sexual orientation and race, ethnicity and racism.

Reporting on sex and gender	N/A

Reporting on race, ethnicity, or other socially relevant groupings	N/A
Population characteristics	N/A
Recruitment	N/A
Ethics oversight	N/A

Note that full information on the approval of the study protocol must also be provided in the manuscript.

Field-specific reporting

Please select the one below that is the best fit for your research. If you are not sure, read the appropriate sections before making your selection.

☒ Life sciences ☐ Behavioural & social sciences ☐ Ecological, evolutionary & environmental sciences

For a reference copy of the document with all sections, see nature.com/documents/nr-reporting-summary-flat.pdf

Life sciences study design

All studies must disclose on these points even when the disclosure is negative.

Sample size	The number of representative cell lines was choses based on our previous study (https://pubmed.ncbi.nlm.nih.gov/33434495/), as described in the main text.
Data exclusions	No replicate data was excluded for this study. For the CRISPRko experiment, we initially considered additional cell line - drug perturbation combinations, however the selected drug concentrations were too low to produce a measurable perturbation.
Replication	For phosphoproteomic profiling, technical replicates were only acquired as described in the main text, due to the large dimensions of the experiment. For the CRISPRko experiment, four different guides per target were generated. No attempts for experimental replication were required or conducted.
Randomization	The phosphoproteomic profiles were generated and measured in different batches, grouped according to cell lines. Within each cell line, data was generated and acquired in a randomized fashion. Normalization was conducted for each cell line separately, thus accounting for potential batch effects. For the CRISPRko experiment, batches were processed according to cell line groups.
Blinding	Experimental blinding was not relevant for this study since our focus was unbiased discovery or large-scale validation.

Reporting for specific materials, systems and methods

We require information from authors about some types of materials, experimental systems and methods used in many studies. Here, indicate whether each material, system or method listed is relevant to your study. If you are not sure if a list item applies to your research, read the appropriate section before selecting a response.

Materials & experimental systems

n/a	Involved in the study	
☒	☐	Antibodies
☐	☒	Eukaryotic cell lines
☒	☐	Palaeontology and archaeology
☒	☐	Animals and other organisms
☒	☐	Clinical data
☒	☐	Dual use research of concern
☒	☐	Plants

Methods

n/a	Involved in the study	
☒	☐	ChIP-seq
☒	☐	Flow cytometry
☒	☐	MRI-based neuroimaging

Eukaryotic cell lines

Policy information about cell lines and Sex and Gender in Research

Cell line source(s)	The cell lines were obtained from ATCC (American Type Culture Collection) (HCT-15: ATCC#CCL-225, LS1034: ATCC#CRL-2158, NCI-H508: ATCC#CCL-253), the Korean Cell Line Bank (KCLB) (SNU-61: KCLB#00061), and the European Collection of Authenticated Cell Cultures (ECACC) (MDST8: ECACC#99011801, HT115: ECACC#85061104).
Authentication	No authentication was conducted after purchase from the vendors.

Mycoplasma contamination	Cell lines used for proteomic analysis were directly obtained from the vendors but not tested for Mycoplasma contamination. For the drug perturbation experiment (336 runs), searching for Mycoplasma peptides identified peptide-spectrum-matches below the confidence threshold (1% PSM FDR). For the baseline profiling experiment (18 runs), small Mycoplasma contamination (5%) for NCI-H508 was observed by this approach. Since this data was only used for auxiliary analysis, which itself only focused on human phosphopeptides, it was not excluded from the study.<br><br>All cell lines were tested for Mycoplasma contamination for the CRISPRko experiment.
Commonly misidentified lines<br>(See ICLAC register)	The six CRC cell lines used in this study (HCT-15, HT115, LS1034, MDST8, NCI-H508, SNU-61) are not listed in ICLAC.
